# The LPAR1 antagonist, PIPE-791 produces antifibrotic effects in models of lung fibrosis

**DOI:** 10.1186/s12931-025-03340-4

**Published:** 2025-08-31

**Authors:** Michael Poon, Kym Lorrain, Alexander Broadhead, Karin Stebbins, Didier Bagnol, Geraldine Edu, Gregory Joseph, Christopher Baccei, Jeffrey Roppe, Thomas Schrader, Lino Valdez, Yifeng Xiong, Austin Chen, Daniel Lorrain

**Affiliations:** Contineum Therapeutics, 3565 General Atomics Court Suite 200, San Diego, CA 92121 USA

## Abstract

**Background:**

Idiopathic pulmonary fibrosis (IPF) is a chronic progressive form of interstitial lung disease (ILD) characterized by significant extracellular matrix deposition, alveolar damage, and tissue remodeling. Antagonists against the G-protein coupled receptor, lysophosphatidic acid receptor 1 (LPAR1) have shown efficacy in lung fibrosis preclinically and clinically. Here, we profile PIPE-791, a small molecule, orally bioavailable LPAR1 receptor antagonist, and show its effectiveness in several lung fibrosis-related contexts.

**Methods:**

In vitro, we used human lung fibroblasts and precision cut lung slices (PCLS) derived from donors with pulmonary fibrosis to test PIPE-791 efficacy in reducing markers of fibrosis. In vivo, we used bleomycin-induced lung fibrosis models to demonstrate PIPE-791 efficacy.

**Results:**

In vitro PIPE-791 reduced LPA-induced collagen expression (IC_50_ 1.1 nM) in human lung fibroblasts. We also show that LPAR1 is elevated in IPF lung tissue and that PIPE-791 significantly reduced several markers of lung fibrosis in PCLS as measured by gene expression and secreted biomarkers. Using in vivo receptor occupancy, we found that PIPE-791 has long association kinetics resulting in a 20-fold increase in potency when dosed 3 versus 24 h prior to radioligand administration. At 3 mg/kg, PIPE-791 was effective in significantly reducing markers of fibrosis and collagen expression in mouse bleomycin models.

**Conclusions:**

We show that PIPE-791 effectively reduces fibrosis and fibrotic markers in vitro and in vivo and that it has slow association and dissociation kinetics. Taken together, our data support clinical testing of PIPE-791 in the context of fibrotic conditions such as IPF.

**Supplementary Information:**

The online version contains supplementary material available at 10.1186/s12931-025-03340-4.

## Introduction

Idiopathic pulmonary fibrosis (IPF) is a chronic, progressive form of interstitial lung disease (ILD) characterized by exaggerated amounts of extracellular matrix, alveolar damage, and tissue remodeling [1, 2]. These lead to significant lung dysfunction, impaired quality of life, and shortened survival. At the cellular level, IPF is a result of aberrant wound-healing following alveolar injury. In response, myofibroblasts produce excess scar tissue resulting in dramatic, detrimental changes in lung structure and function. Current standard of care for IPF include two pharmacotherapies, pirfenidone and nintedanib. While classified as an antifibrotic, perfenidone’s mechanism of action is unknown. Nintedanib, a pan-tyrosine kinase inhibitor, blocks myofibroblast activation which leads to fibrosis [3].

Aberrant lysophosphatidic acid (LPA) signaling via LPAR1 is linked to the etiology of IPF [4–6]. LPA is derived from the conversion of lysophosphatidylcholine by autotaxin and normally serves to promote wound healing. Specifically, LPA-mediated activation of LPAR1 and its downstream signaling contribute to inflammation and fibrotic mechanisms. In disease contexts such as IPF, patients exhibit elevated levels of LPA in bronchoalveolar lavage fluid leading to abnormal LPAR1 activation [5]. As a result, LPAR1 inhibition has garnered substantial interest as a target to address fibrotic diseases.

LPAR1 is a G-protein coupled receptor that belongs to a six-member family of LPA receptors, each associated with several, sometimes overlapping, signaling mechanisms. These effects include cell proliferation and differentiation, cytoskeletal regulation, chemotaxis, inflammation, and cell adhesion, many of which have been shown to contribute to fibrosis [7]. LPAR1 is expressed in numerous cell types, including macrophages and fibroblasts [8–10]. Upon activation, LPAR1 induces inflammation and fibrosis in various tissues including lung, liver, and kidney. After injury (e.g., bleomycin), LPA levels increase, activating LPAR1, promoting a profibrotic state [5]. Specifically, LPAR1 activation increases accumulation of extracellular matrix (ECM) components, inflammatory signaling via NF-κB and c-jun N-terminal kinase, and expression of chemokines and inflammatory cytokines, such as interleukin-6 (IL-6) [6].

There is abundant evidence supporting LPAR1 antagonism for treating various organ fibroses, including lung. As such, we profiled PIPE-791, a potent, small molecule LPAR1-selective antagonist with uniquely favorable pharmacological kinetics, in various lung fibrosis contexts. We show that PIPE-791 mitigates chemotaxis and collagen production (two prominent features in the etiology of IPF) in cultured primary human lung fibroblasts. We also show that LPAR1 protein levels are elevated in IPF lung and, using precision cut lung slice (PCLS) cultures from donors with pulmonary fibrosis (PF), show that PIPE-791 reduces several key fibrotic markers as assessed by gene expression and secreted biomarkers of fibrogenesis and lung remodeling. To evaluate in vivo lung receptor occupancy, we developed an LPAR1 radioligand [^3^H]-PIPE-497 and show dose dependent displacement following oral administration of PIPE-791. Importantly, we observe 24-hour receptor coverage after a single oral dose. Functionally, PIPE-791 dose-dependently inhibits an LPA-induced histamine response in blood–an effect that persists 24 h following a single oral administration, corresponding with prolonged LPAR1 receptor occupancy. Using this information to guide dose selection and frequency, we show that PIPE-791 is effective both prophylactically and therapeutically at reducing lung collagen in bleomycin-induced lung fibrosis models. Lastly, we show that PIPE-791 reduces IL-1β release in rodent and human alveolar macrophages, highlighting a possible role for alveolar macrophages in the etiology of lung fibrosis.

## Results

### PIPE-791 is selective for human LPAR1

PIPE-791 is an LPAR1 antagonist that exhibits distinct slow association and dissociation kinetics with little off-target activity. We had previously assessed selectivity against LPAR2 and 3 [11]. To gain additional insight into LPA receptor selectivity, we tested and compared PIPE-791 potency to LPAR2-6, striving to maximize biological equivalence between receptors by using the same cell type, overexpression method, and functional endpoint. Human LPAR1-6 plasmids were each transiently transfected into HEK293T cells and the EC_80_ (effective concentration producing 80% maximal effect) for LPA activation empirically determined. Selectivity was then tested in a concentration response paradigm using a 3 h preincubation followed by LPA-induced calcium mobilization. In this assay, PIPE-791 had a functional IC_50_ of 8 nM, and fold selectivity against LPA2 through 6 of 58x, 50x, 76x, 14x, and 101x, respectively (Table 1).

**Table 1:** PIPE-791 selectivity against LPA 2–6

	LPA1	LPA2	LPA3	LPA4	LPA5	LPA6
LPA EC_80_ (µM)	5	5	10	10	1	13
IC_50_ (nM)	8	463	398	607	114	805
Selectivity	-	58x	50x	76x	14x	101x

Table of PIPE-791 selectivity using a calcium mobilization assay in HEK293T cells transiently transfected with plasmids encoding one of LPA1-6. Values are empirically determined LPA EC_80_ concentration used for stimulation, PIPE-791 potency values, and fold selectivity versus LPA1

Earlier generation LPAR1 antagonists have suffered from off-target inhibition of the bile salt export pump (BSEP), which can result in hepatobiliary toxicity [12]. When assayed for activity against BSEP, PIPE-791 showed minimal inhibition, with an IC_50_ of 24.4 µM, 3000-fold higher than its LPAR1 IC_50_. Using a sandwich-cultured human hepatocyte assay which contains functioning efflux transporters for assessing general and cholestatic liver injury (cholestatic drug-induced liver injury assay or c-DILI), 30 µM PIPE-791 showed neither an appreciable increase in lactate dehydrogenase (an indicator of cell death), nor a decrease in ATP content (a cell viability measure, Supp Fig S1) in the presence or absence of a toxic concentration of bile acids.

### PIPE-791 inhibits chemotaxis and collagen production in primary human lung fibroblasts

**Fig. 1:**
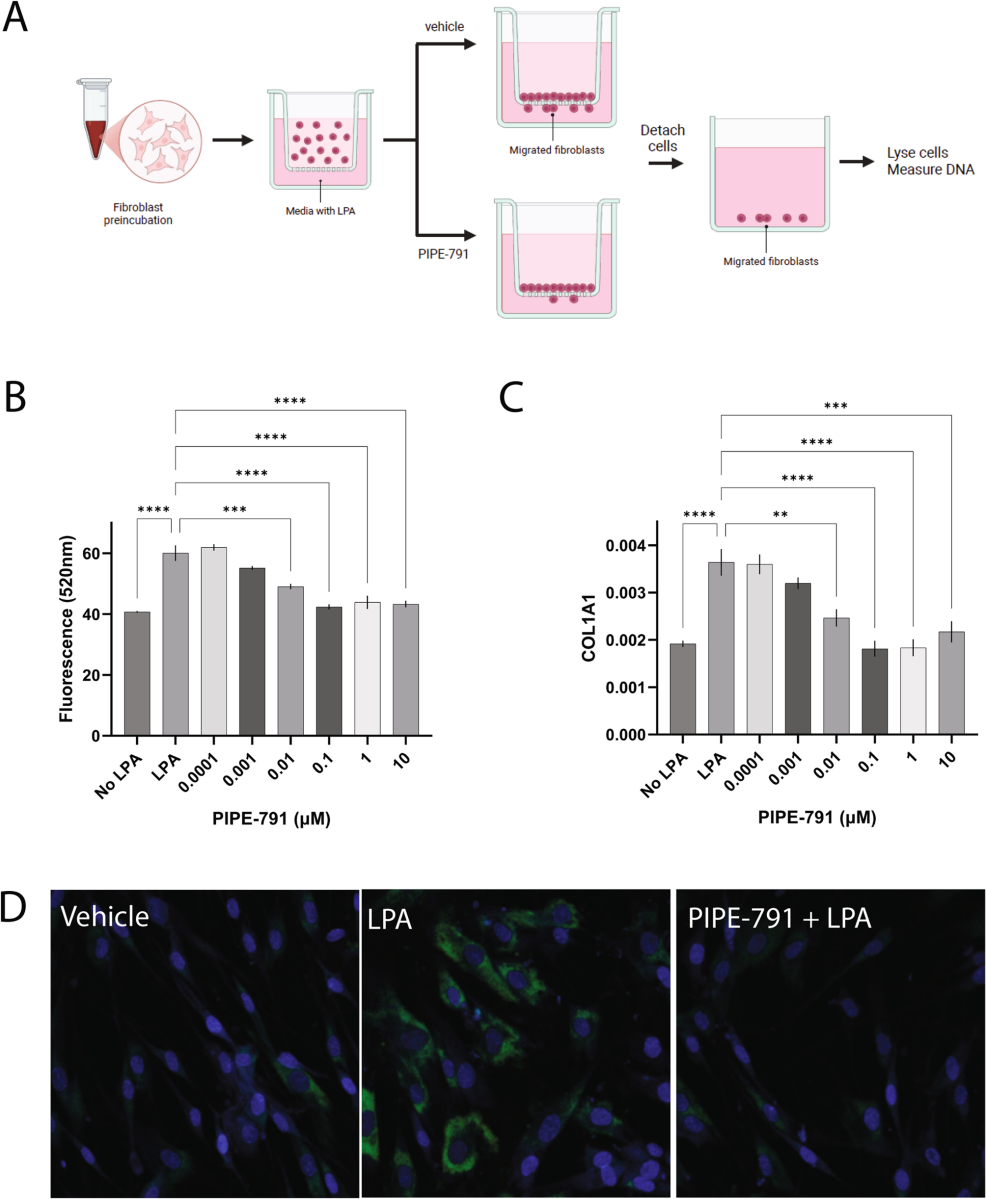
PIPE-791 inhibits collagen expression and chemotaxis in primary adult human lung fibroblasts. **A**. Diagram outlining the chemotaxis assay: human lung fibroblasts are incubated with PIPE-791 and plated onto a Transwell culture insert with LPA in bottom chamber as a chemoattractant. Fibroblasts migrate through the pores and along the bottom surface. Cells on the bottom surface are detached, lysed and the lysate analyzed for DNA content with DAPI. **B**. Concentration dependent inhibition of LPA-induced chemotaxis as measured by DAPI fluorescence (A_520_ nm). PIPE-791 inhibits chemotaxis at an IC_50_ of 1.5 nM (n=3, ** P < 0.01, *** P < 0.001, **** P < 0.0001, one-way ANOVA, error bars SEM). **C**. Concentration dependent inhibition of LPA induced COL1A1 expression in fibroblasts. PIPE-791 inhibits COL1A1 induction with an IC50 of 1.1 nM (n=3, ** P < 0.01, *** P < 0.001, *** P < 0.0001, one-way ANOVA, Newman-Keuls, error bars SEM). **D**. Representative images of primary human lung fibroblasts treated with vehicle, LPA, or PIPE-791 and LPA and stained for COL1A1 (green) and Hoechst (blue). Each micrograph is 100 µm width

During lung fibrosis, activated fibroblasts migrate to the site of injury where they produce and secrete ECM components, like collagen. Both phenomena have been shown to be inhibited by other LPAR1 small molecule antagonists or in LPAR1 knockout mice [4, 5]. We sought to verify the impact of antagonizing LPAR1 with PIPE-791 on lung fibroblast chemotaxis and collagen production in vitro. Primary adult human lung fibroblasts were incubated with PIPE-791 and added to a chemotaxis chamber plate using LPA as a chemoattractant (Fig. 1 A). We observed that PIPE-791 inhibited fibroblast chemotaxis at an IC_50_ 1.5 nM (Fig. 1B). To induce collagen production, fibroblasts were incubated with PIPE-791 then LPA added to induce fibroblast differentiation and assessed for COL1A1 expression by immunocytochemistry. PIPE-791 inhibited COL1A1 at an IC_50_ of 1.1 nM (Fig. 1 C and D). In this assay, a different small molecule LPAR1 antagonist, BMS-986278 inhibited collagen expression with a potency of 30.6 nM (Supp Fig. 2). These chemotaxis and collagen data are in good agreement with each other and consistent with previous human fibroblast data [11].

## PIPE-791 inhibits myofibroblast transformation

**Fig. 2:**
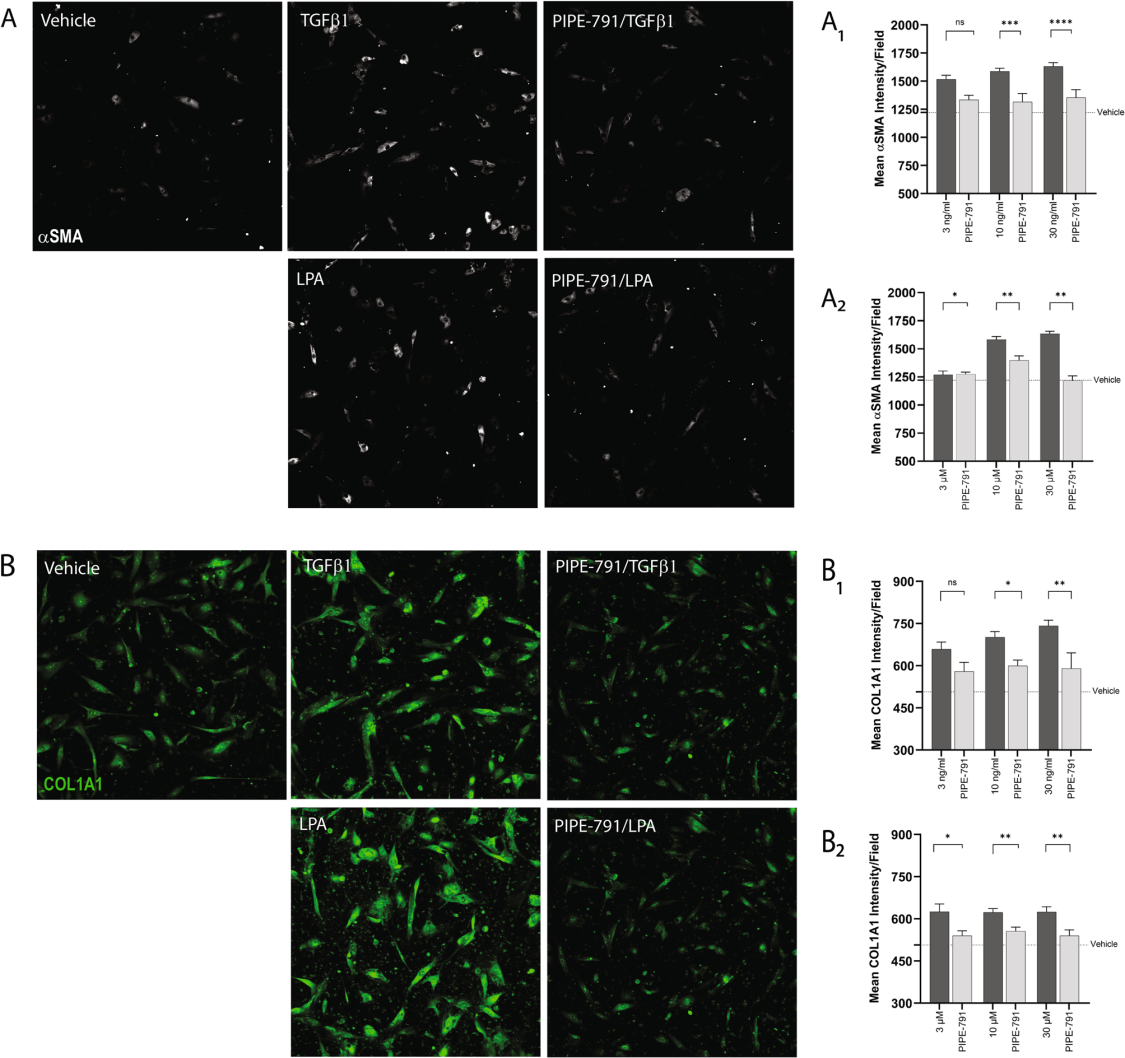
PIPE-791 inhibits TGFβ1 or LPA-induced myofibroblast transformation. Human adult fibroblasts were treated and co-stained for αSMA and COL1A1. **A**. Representative images of fibroblasts stained with αSMA (white) after treating with vehicle, TGFβ1, or PIPE-791/TGFβ (top row) or LPA, PIPE-791/LPA. A_1_. PIPE-791 (1 μM) reduced αSMA at all concentrations of TGFβ1 or (A_2_) LPA tested. **B**. Representative images of primary human lung fibroblasts stained for COL1A1 (green) after treating with vehicle, TGFβ1, or PIPE-791/ TGFβ (top row), or LPA, PIPE-791/LPA. B_1_. PIPE-791 (1 μM) reduced COL1A1 expression at all concentrations of TGFβ1 or (B_2_) LPA tested (*n*  >  5, * *P* < 0.05, ** *P* < 0.01, *** *P* < 0.001, **** *P* < 0.0001, t-test, error bars SEM)

Activation of fibroblasts by TGFβ1 or LPA results in their transition to a myofibroblast phenotype. This is characterized by aSMA-expression resulting in the subsequent deposition of ECM components. TGFβ1 leads to the induction of numerous cytokines, including IL-6 [13] Interestingly, in fibroblasts, IL-6 has been found to induce fibroblast expression of autotaxin and vice versa, resulting in rapid, amplified LPA synthesis [14]. In light, we were curious whether PIPE-791 could prevent myofibroblast transformation following direct LPA addition or indirect LPA synthesis via TGFβ1. Cultured primary human lung fibroblasts were treated with PIPE-791, stimulated, then analyzed for αSMA expression. Addition of either TGFβ1 or LPA resulted in a significant increase in αSMA expression indicating transformation into myofibroblasts. After either insult, αSMA expression was significantly inhibited in the presence of PIPE-791 (Fig. 2 A). Treatment with PIPE-791 also significantly inhibited COL1A1 induction after either insult (Fig. 2B).

### LPAR1 protein expression is higher in IPF lung tissue

While *LPAR1* mRNA has been shown to be elevated in IPF donor lung tissue and in preclinical pulmonary fibrosis models, scant data around protein expression (particularly in human tissue) has been lacking [15]. This is, in large part, due to a lack of adequate antibodies against LPAR1. Radiolabeled small molecule probes, which are also amenable for human PET imaging, have provided a means of detecting LPAR1. For example, using a PET probe for LPAR1, [^18^F]-BMS-986327, LPAR1 binding was detected in the lungs of naïve non-human primate and in rats treated with bleomycin and was displaceable by a cold, unlabeled LPAR1 antagonist [16].

**Fig. 3:**
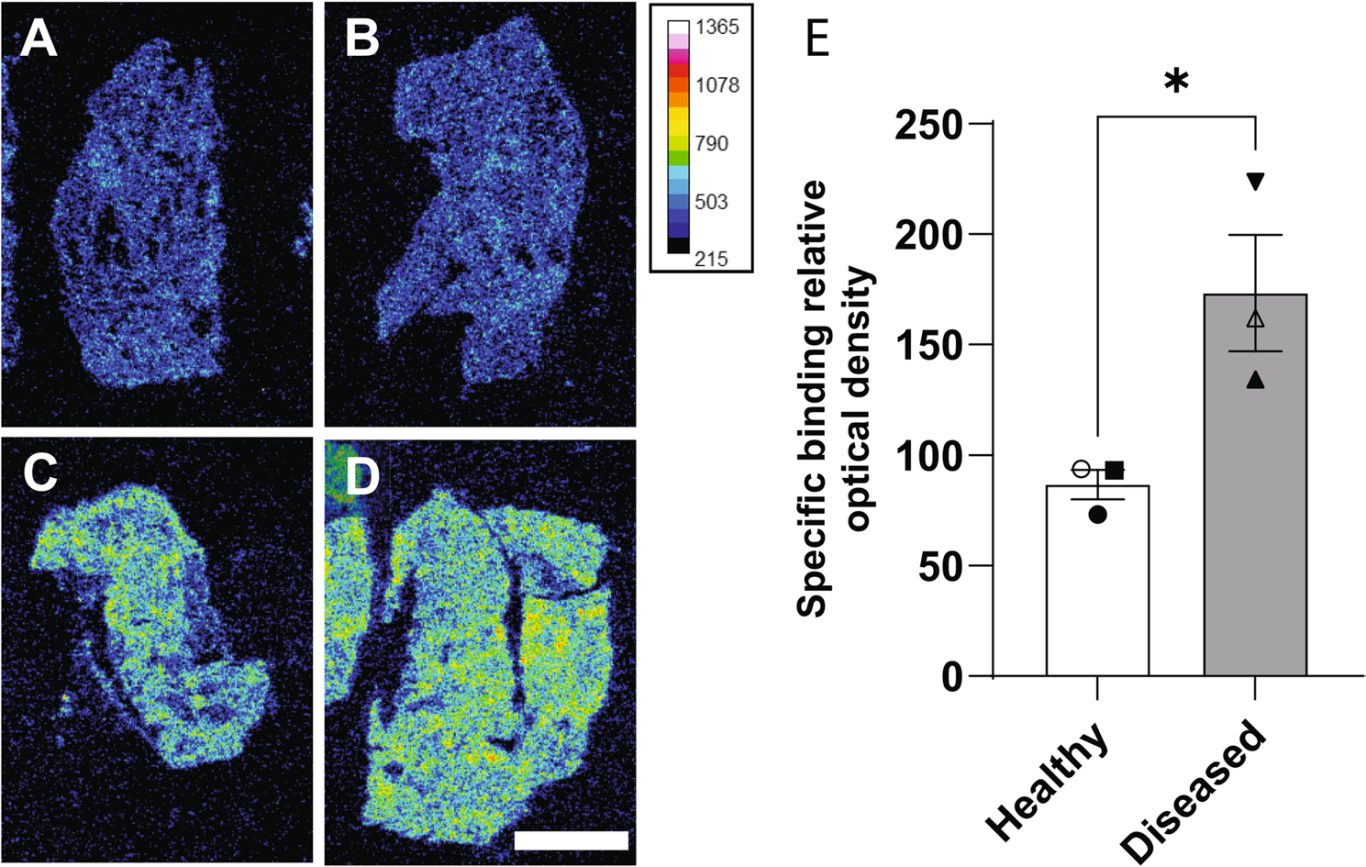
LPAR1 is more highly expressed in diseased lung tissue. **A-D**. Representative images illustrating the total binding of [^3^H]-PIPE-497 (20 nM) on human lung sections from healthy (**A**, **B**) and diseased lungs (**C**, **D**). Intensity scale of the relative optical density is shown. Scale bar = 0.5 mm. **E**. Bar graph with individual values showing increased in LPAR1 specific binding signal in diseased compared to healthy donors. (n = 3 for healthy and diseased tissue, * P < 0.05, t-test, error bars SD). Symbols correspond to donors in Additional File 1 as follows: ⬤ Donor A, ◯ Donor B, ⬛ Donor C, ▲ Donor D, △ Donor E, ▼Donor F

Similarly, we identified, characterized, and radiolabeled a small molecule LPAR1 antagonist to yield [^3^H]-PIPE-497 (Supp Fig. 3, Supp Table 1). Autoradiography was performed on sections from normal and IPF donor lung tissue and LPAR1 signal obtained by subtracting non-specific from specific binding (Fig. 3A-D, Supp Fig. 4, donor information in Additional File 1). In this manner, we observed 2-fold increase in expression of LPAR1 in IPF donor lungs compared to healthy donors, confirming the *LPAR1* transcript data, and providing important evidence that LPAR1 protein is also higher in the diseased state (Fig. 3E). We also observed 1.7-fold increase in LPAR1 in mouse lung sections by ex vivo autoradiography, 3 days following intratracheal injection of bleomycin (Supp Fig S5).

### PIPE-791 reduces mRNA expression of fibrotic markers in human precision cut lung slice

Human precision cut lung slice cultures have emerged as a physiologically relevant ex vivo model for studying compound efficacy in the context of fibrosis [17]. In normal PCLS treated with pro-fibrotic factors like TGFβ and TNFα, while there is induction of fibrotic gene markers, no actual fibrotic remodeling is observed. Further, while possible to induce early fibrotic-like changes in normal PCLS with various cocktails, the complex interplay between the different factors are still being understood [18]. Here, we chose to focus our efforts on PCLS from donors diagnosed with pulmonary fibrosis, the underlying question being whether inhibiting LPAR1 in these slices with PIPE-791 would impact the expression of fibrosis-related mRNAs.

**Fig. 4:**
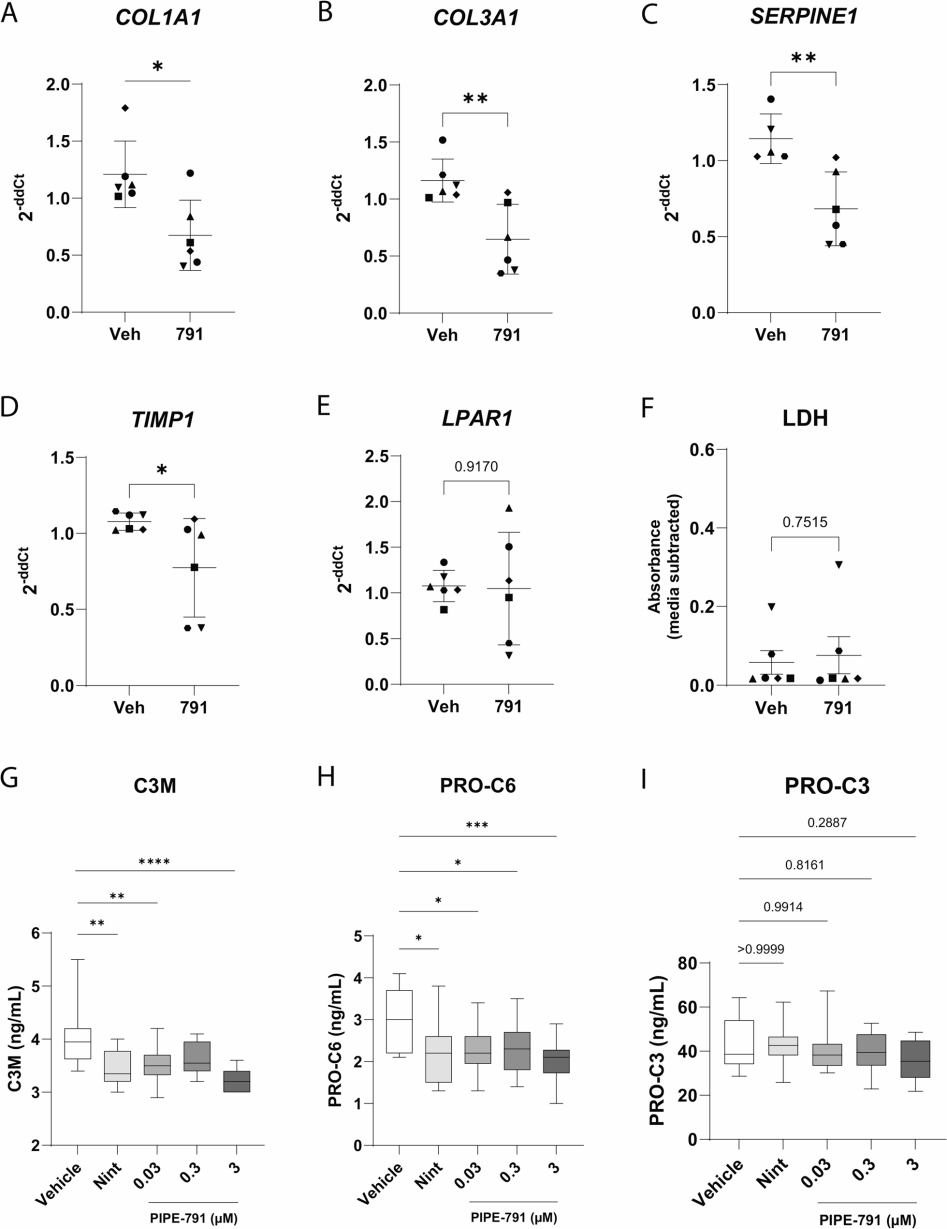
PIPE-791 reduces expression of fibrotic markers in human PCLS. **A-F**. Six day incubation of PIPE-791 with PCLS from donors with pulmonary fibrosis decreases COL1A1 expression (**A**), COL3A1 (**B**), SERPINE1 (**C**), and TIMP1 (**D**) (n = 6 donors, * P < 0.05, ** P < 0.01, t-test, error bars SD). **E**. PIPE-791 does not significantly change LPAR1 expression (n = 6, n.s. not significant, t-test). **F**. PIPE-791 does not impact slice health compared to vehicle treated PCLS as measured by LDH in the media (n = 6, n.s. not significant, t-test). Symbols correspond to donors in Additional File 1 as follows: Donor G; ■ Donor H; ▲ Donor I; ◆ Donor J; ⬣ Donor K; ▼ Donor L. **G-I**. Forty-eight hour incubation of nintedanib or 3 concentrations of PIPE-791 (0.03, 0.3, or 3 µM) with fresh PCLS from donors with pulmonary fibrosis results in significant inhibition of secreted C3M (**G**) and Pro-C6 (**H**). Pro-C3 (**I**) did not show a significant decrease with nintedanib or PIPE-791 (n = 12, two donors, two lung regions in triplicate, * P < 0.05, ** P < 0.01, *** P < 0.001, **** P < 0.0001, one-way ANOVA, Dunnett’s post-hoc, error bars are SD). Donor information can be found in Additional File 1

PCLS from six separate donors were removed from cryopreservation, recovered for 24 h in culture, then treated with vehicle or PIPE-791 for 6 days, similar to methods described in Decaris et al., [19]. Slices were snap frozen and processed for transcript analysis by quantitative PCR. After treatment with PIPE-791, we observed significantly less expression of *COL1A1*, *COL3A1*, *SERPINE1*, and *TIMP1* compared to vehicle treated PCLS (Fig. 4A-E). *LPAR1* levels were not significantly affected by PIPE-791 (Fig. 4 F). Further, PIPE-791 had no significant effect on slice health as measured by LDH (Fig. 4G). Concurrently, we evaluated two well-established, clinical stage antifibrotic mechanisms in this setting: an αvβ1/6 inhibitor (bexotegrast, PLN-74809) and a PDE4B inhibitor (nerandomilast, BI 1015550). With both mechanisms, we saw significantly less *COL1A1* compared to vehicle treated slices. *TIMP-1* expression was also significantly lower in the presence bexotegrast (Supp Fig S6 and S7) [19, 20].

In normal, non-fibrotic PCLS cultures, PIPE-791 did not alter the expression of any of the fibrotic marker transcripts tested (Supp Fig S8).

### PIPE-791 inhibits production of biomarkers in PCLS cultures

Translationally, biomarker-based strategies for assessing aberrant extracellular matrix deposition and remodeling in the context of disease pathogenesis and compound efficacy have become increasingly relevant. For the PCLS gene expression studies, slices were preserved through slow cryogenic freezing of tissue. While this affords experimental flexibility, some physiological relevance is lost over the course of cryostorage and recovery (e.g., immune cell loss). To circumvent this, fresh PCLS from two donors with pulmonary fibrosis were generated and immediately placed into culture. For this experiment, well-established, secreted fibrotic markers that encompass fibrogenesis and tissue remodeling were measured: C3M (a marker of collagen III degradation and an indicant of ECM remodeling and tissue turnover), Pro-C3 (a neo-epitope fragment of collagen III and a marker of active fibrogenesis and fibrosis progression), and Pro-C6 (a neoepitope fragment of COLVIA3 and a marker of remodeling and wound healing). Slices were treated for 48 h with vehicle, nintedanib, or PIPE-791 as a positive control [21]. Media was then removed and analyzed for biomarker concentration.

After a 48 h incubation with nintedanib, we observed a significant reduction of secreted C3M, but not the neoepitope Pro-C3, consistent with Hesse et al., 2022 (Fig. 4G) [21]. Nintedanib also reduced the neoepitope Pro-C6, consistent with what has been observed in IPF patients (Fig. 4H) [22]. With PIPE-791, levels similar or superior to nintedanib were achieved. Specifically, we observed a reduction in C3M secretion with PIPE-791 at all concentrations tested, with significance observed at 0.03 and 3 µM (Fig. 4G). We also observed significant reduction in secreted Pro-C6 at all concentrations of PIPE-791 tested (Fig. 4H). Like nintedanib, however, we did not observe a significant decrease in Pro-C3 with PIPE-791 (Fig. 4I).

### PIPE-791 displays extended in vivo plasma exposure

**Fig. 5:**
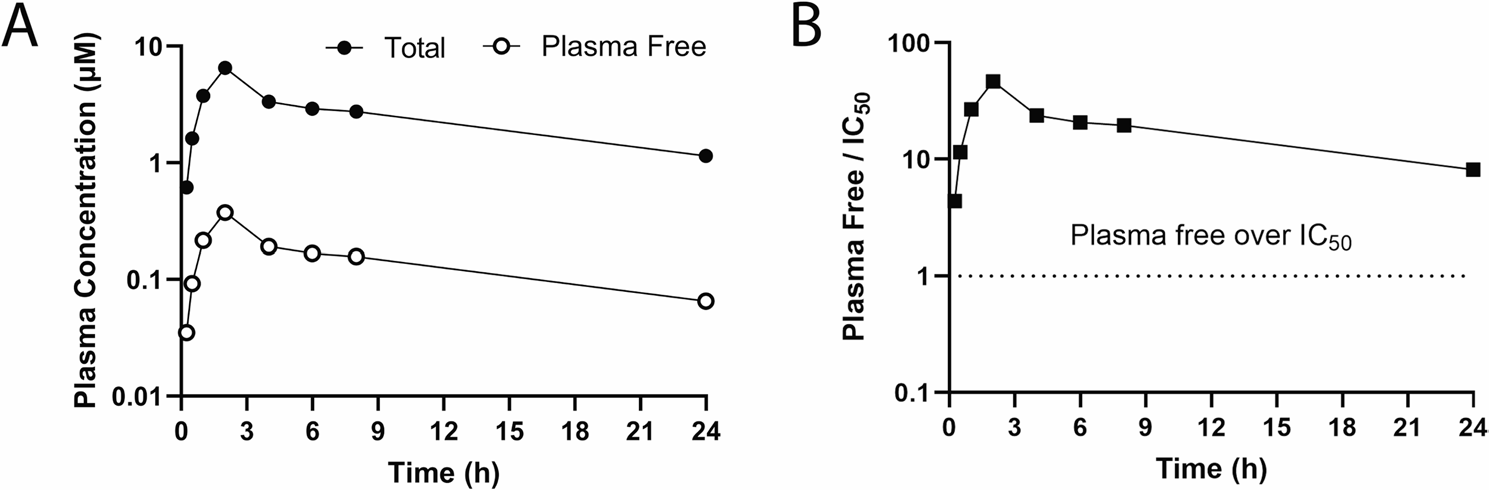
PIPE-791 has extended plasma exposure in rat. **A** Rats were orally dosed with 10 mg/kg PIPE-791 and plasma concentrations taken at 0.25, 0.5, 1, 2, 4-, 6-, 8-, and 24-hours post dose. Total PIPE-791 concentration (solid circles) and free concentrations (after correcting for a rat plasma fraction unbound of 0.057, open circles) shown, *n* = 2. **B** Graph depicting PIPE-791 plasma free (unbound) concentration as a function of time over the in vitro calcium mobilization IC_50_. At 10 mg/kg, the free PIPE-791 concentration in plasma is still well above its LPAR1 IC_50_ at the last timepoint measured (24 h).

To determine the pharmacokinetic profile of PIPE-791 in vivo, we measured rat plasma concentrations of PIPE-791 at multiple timepoints following an oral dose of 10 mg/kg. We found that even at 24 h post-dose, plasma unbound PIPE-791 concentrations exceeded the in vitro calcium mobilization IC_50_ (Fig. 5 A and B). This distinct, long-lived pharmacokinetic profile would support a dosing frequency less than that of other LPAR1 antagonists described in the literature. In comparison, the plasma unbound concentration of admilparant (BMS-986278), an LPAR1 antagonist being evaluated in Phase 3 for the treatment of IPF and progressive pulmonary fibrosis (PPF) at oral doses of 60 and 120 mg twice a day (BID), was only above its measured IC_50_ (80.4 nM ×/÷ 1.58, *n* = 6) for 4 h when dosed orally in rats at 10 mg/kg (Supp Fig S9).

### In vivo LPAR1 lung receptor occupancy

**Fig. 6:**
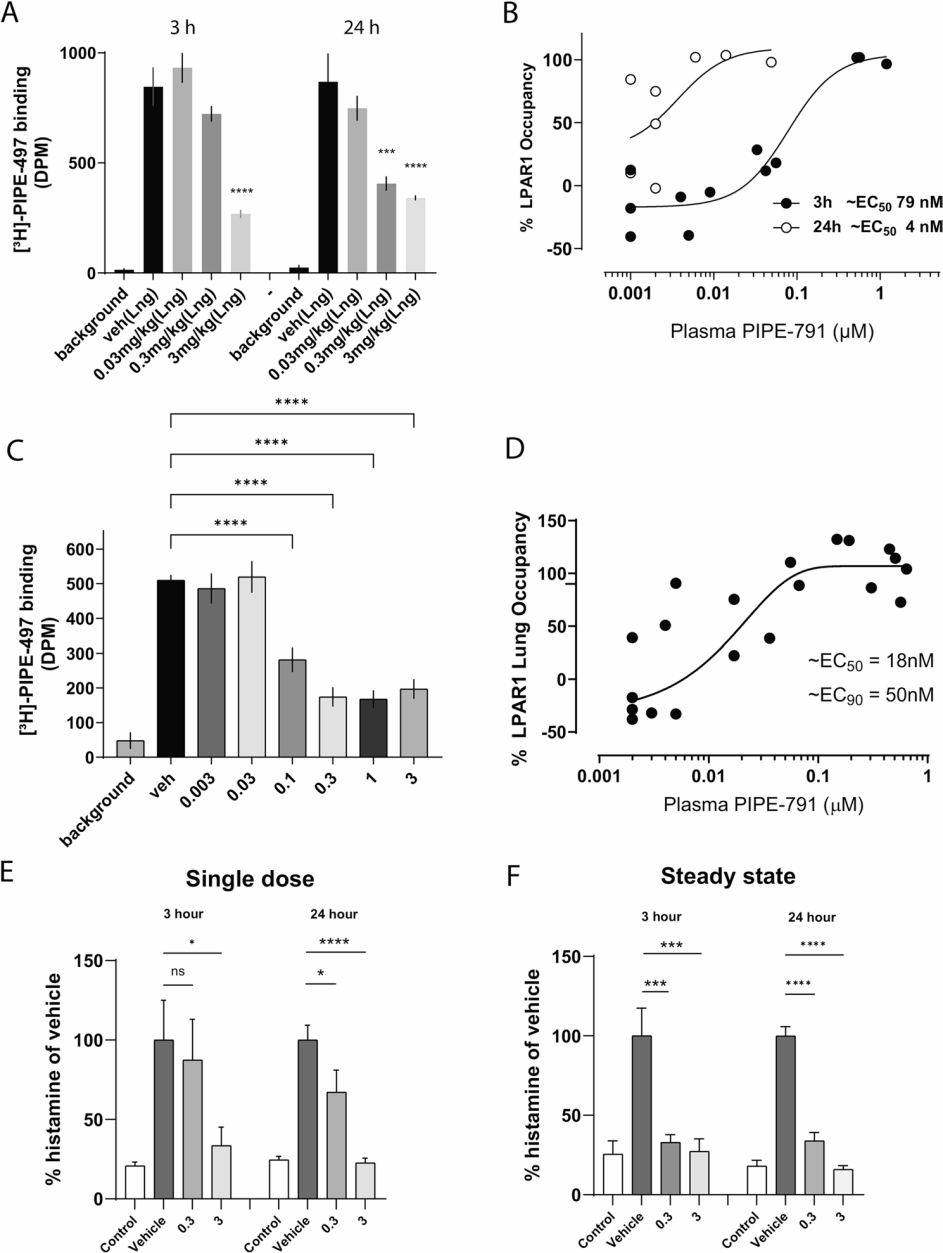
PIPE-791 has long receptor dissociation kinetics and inhibits mast cell histamine release in vivo. **A** Mice were orally dosed with PIPE-791 followed by intravenous injection of the LPAR1 selective radiotracer [^3^H]-PIPE-497 at 3 or 24 h post dose and lung collection. Significant radiotracer displacement (LPAR1 receptor occupancy) was observed at 3 mg/kg at 3 h, whereas significant occupancy was observed at both 0.3 and 3 mg/kg at 24 h (*n* > 4, ANOVA with Newman-Keuls, *** *P* < 0.001, **** *P* < 0.0001, error bars SEM). **B** Values plotted as total plasma concentration versus percent occupancy highlighting increase in EC_50_ from 79 nM at 3 h to 4 nM at 24 h, 3 h (solid circles) and 24 h (open circles, error bars SEM). **C** Mice were orally dosed PIPE-791 for 4 days, once a day to simulate steady state conditions. Lung occupancy was measured by i.v. injection of [^3^H]-PIPE-497. Lung receptor occupancy gave an ED_50_ of 0.1 mg/kg (*n* > 5, except background *n* = 4, **** *P* < 0.0001, One way ANOVA, with Tukey’s, error bars SEM). **D** Based on total plasma concentrations, the calculated EC_50_ and EC_90_ were 18 nM and 50 nM, respectively. **E** Mice were orally dosed with PIPE-791 at 0.3 or 3 mg/kg then challenged with intravenous LPA 3–24 h later. Blood histamine levels were analyzed. Significant reduction in histamine was observed at 3 mg/kg at 3 h and at both 0.3 and 3 mg/kg at 24 h (*n* > 4 for all groups except vehicle at 3 h *n* = 2; ANOVA, uncorrected Fisher’s LSD, * *P* < 0.05, **** *P* < 0.0001, error bars SEM). **F** Mice were dosed with PIPE-791 for 4 days to achieve steady-state followed by LPA challenge and blood histamine collection at 3 and 24 h post last dose. Significant reduction at both 0.3 and 3 mg/kg at both 3 and 24 h (*n* = 6, ANOVA with Newman-Keuls, *** *P* < 0.001, **** *P* < 0.0001, error bars SEM)

We next determined LPAR1 receptor occupancy at a given dose and time by utilizing [^3^H]-PIPE-497, which we found to be suitable for in vivo receptor occupancy studies (Supp Fig S3, Supp Table 1) [11]. In lung, when dosed 3 h prior to intravenous injection of the radioligand, PIPE-791 exhibited an ED_50_ of 0.5 mg/kg. However, when dosed 24 h prior to radiotracer, we observed a lower ED_50_ of 0.09 mg/kg. From the quantified free PIPE-791 plasma concentration, these data corresponded to EC_50_ values of 79 nM at 3 h and 4 nM at 24 h (Fig. 6A-B). These data reveal an unexpected advantage for PIPE-791, specifically, its long binding kinetics. This, in concert with its potency and excellent oral PK, provides PIPE-791 the advantage of a low efficacious dose and a less frequent dosing regimen. We also note that following repeat dosing to simulate steady state (once per day for 4 days), 3 h after the last dose the ED_50_ was 0.1 mg/kg and the corresponding EC_50_ was 18 nM, similar to the single dose, 24 h value. (Fig. 6 C and D). These data demonstrate that PIPE-791 has remarkably long binding kinetics, consistent with the kinetics observed in other tissues, such as brain [11].

### PIPE-791 inhibits in vivo LPA-induced mast cell histamine release

After stimulation with LPA, mast cells rapidly release histamine in an LPAR1-dependent manner. As such, several groups have utilized LPA-induced histamine release as an LPAR1-sensitive endpoint [23–26]. Mice were orally administered PIPE-791 at 0.3 or 3 mg/kg, then injected intravenously with 300 µg LPA 3–24 h later. Two minutes after LPA challenge, blood was collected and serum was analyzed for histamine. At either 3–24 h, significant blockade of histamine release was observed at 3 mg/kg. At 0.3 mg/kg, significance was observed at 24 h but not at 3 h (Fig. 6E). This suggests that with time, PIPE-791 increasingly occupies more of the LPAR1 receptor, consistent with our receptor occupancy data and the notion of gradual receptor association kinetics. We also tested steady-state conditions where PIPE-791 was dosed orally for 4 consecutive days, followed by LPA stimulation 3–24 h after the final dose. In this case, significant efficacy at 0.3 and 3 mg/kg was observed at both timepoints (Fig. 6 F).

### PIPE-791 reduces fibrotic markers in a mouse IT bleomycin model

We tested the potential for PIPE-791 to reduce markers of fibrosis in a 14-day lung intratracheal bleomycin model. Intratracheal instillation of bleomycin results in acute alveolar cell damage, inflammation, and subsequent tissue remodeling and is a widely used preclinical IPF model [4]. Here, mice were treated with 0.03, 0.3, or 3 mg/kg PIPE-791, then bleomycin administered intratracheally to induce lung fibrosis. PIPE-791 dosing was maintained orally, once a day, for 14 additional days.

**Fig. 7:**
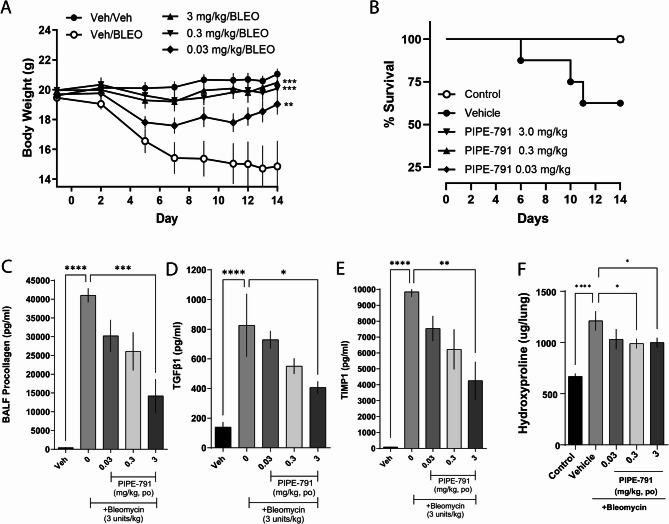
PIPE-791 reduces fibrotic markers in an intratracheal bleomycin model. **A** Mice treated with PIPE-791 show dose dependent body weight improvement compared to vehicle/bleomycin (Veh/BLEO) treated mice (*n* = 8, deaths excluded, one way ANOVA at day 14, ** *P* < 0.01, *** *P* < 0.001, error bars are SEM). **B** Survival plot of study over time. No deaths were observed in the PIPE-791 treated groups. **C** BAL fluid was collected at study termination and analyzed for fibrotic markers. PIPE-791 significantly inhibited several markers in the presence of bleomycin in a dose dependent fashion including procollagen, (**D**) TGFβ1, and (**E**) TIMP1 (*n* = 8, deaths excluded, ANOVA with Newman-Keuls, * *P* < 0.05, ** *P* < 0.01, *** *P* < 0.001, **** *P* < 0.0001, error bars SEM). **F** PIPE-791 reduced collagen amounts in lung tissue (*n* = 8, excluding deaths, one-way ANOVA with Newman Keuls, * *P* < 0.05, **** *P* < 0.0001, error bars SEM)

Treatment with bleomycin resulted in significant body weight loss. By day 14, naïve mice had an average body mass of 21.05 g. Bleomycin treated mice had an average body weight of 14.86 g, a 29% loss. In comparison, bleomycin mice dosed with PIPE-791 showed significant dose dependent improvements in body weights: at 0.03 mg/kg PIPE-791, mice showed a 28% improvement over bleomycin alone. At 0.3 mg/kg mice showed a 41% improvement and at 3 mg/kg, a 38% improvement (Fig. 7 A). Notably, in the bleomycin/vehicle treated group, 3 mice died, whereas PIPE-791 improved survival with no mice lost in any of the PIPE-791 dosed groups (Fig. 7B).

Bronchoalveolar lavage (BAL) fluid was collected and tested for validated markers of lung fibrosis: procollagen, TGFβ, and TIMP1 [4]. In all cases, dose-dependent decreases in these markers were observed. At 3 mg/kg PIPE-791, we saw significant reduction of all markers, specifically, a 65% reduction in procollagen, a 51% reduction in TGFβ, and a 57% reduction in TIMP1 compared to bleomycin/vehicle treated mice (Fig. 7C-E).

Lung tissues were collected and processed for hydroxyproline [4, 5]. Here, bleomycin induced an 81% increase in tissue collagen. In the presence of PIPE-791, we observed a significant 41% reduction in hydroxyproline at 0.3 mg/kg and a similar 39% reduction in the 3 mg/kg groups. While mice treated with 0.03 mg/kg PIPE-791 showed a trending decrease in collagen, the values did not reach statistical significance (*p* = 0.077, Fig. 7 F).

### Therapeutic dosing of PIPE-791 reduces lung collagen in a systemic bleomycin-induced lung fibrosis model

Systemic, subcutaneous administration of bleomycin is a clinically relevant model of chronic lung injury that shares many features also observed in IPF patients. In comparison to intratracheal delivery, which can lead to variable distribution and high mortality, systemic delivery provides a more uniform distribution of fibrosis, with fibrosis seen interstitially and along the subpleural lining of the lung [27]. Subcutaneous bleomycin delivery also results in mononuclear cell infiltration, endothelial damage, and macrophage infiltration [28].

Mice were injected subcutaneously with bleomycin (0.1 units/day), on a schedule of 5 days on/2 days off to induce fibrosis. PIPE-791 was dosed therapeutically at 3 mg/kg orally, once a day, commencing 7 days after the first bleomycin injection. Similarly, a clinical stage LPAR1 antagonist, BMS-986020, was dosed therapeutically, at 30 mg/kg orally, twice a day. Dosing was maintained until study end, at which point lung tissues were collected. The right lobe was snap frozen and processed for collagen analysis for hydroxyproline; the left lobe was sectioned and stained with Masson’s trichrome stain for histological assessment of tissue collagen.

**Fig. 8:**
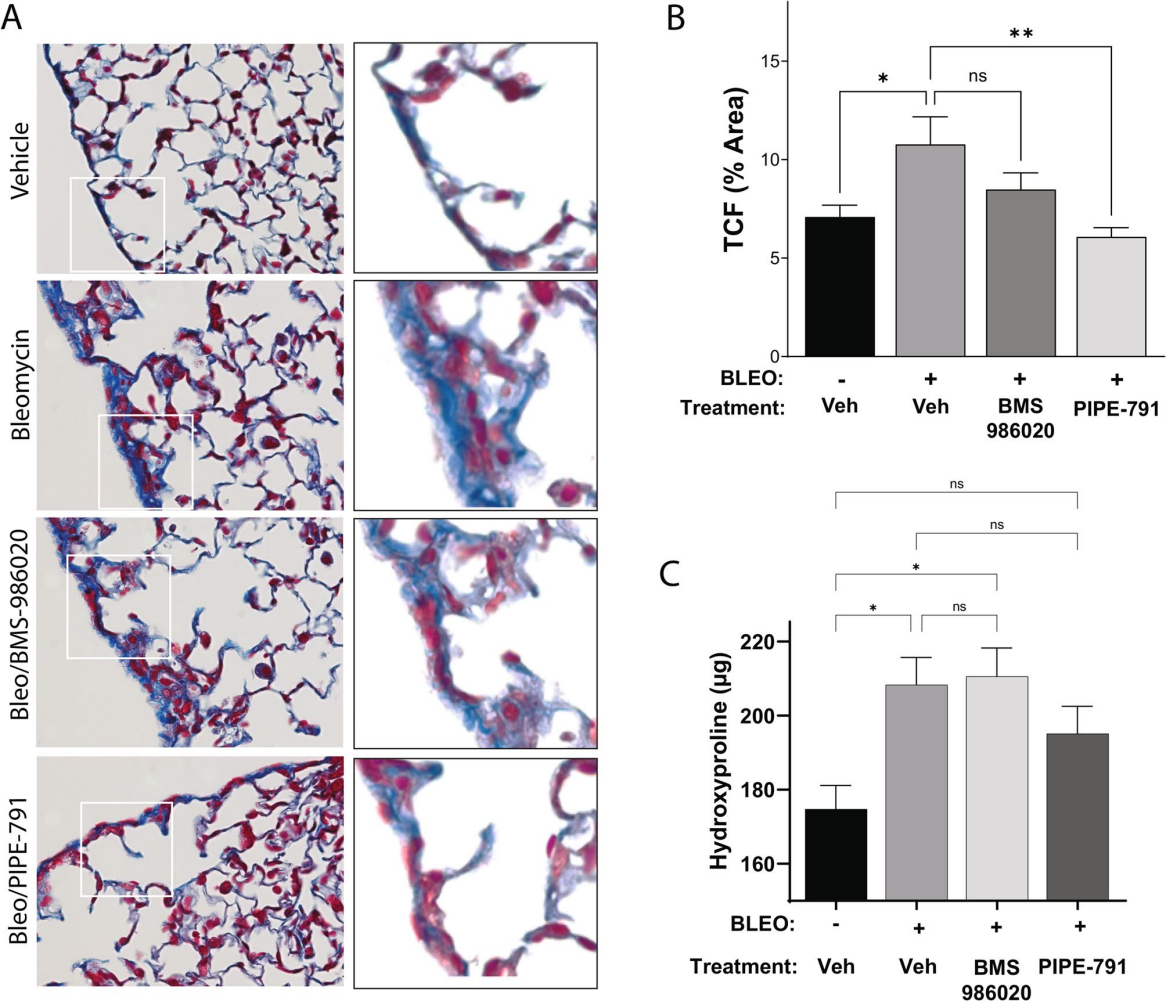
Therapeutic dosing of PIPE-791 reduces fibrotic markers in a systemic bleomycin model. **A** Representative trichrome stained images of vehicle/vehicle, bleomycin/vehicle, bleomycin/BMS-986020, and bleomycin/PIPE-791 treated mice (blue, collagen). BMS-986020 was used as a positive control LPAR1 antagonist. Right column is magnified image of region in white box. **B** PIPE-791 significantly reduces lung tissue collagen fraction as assessed histologically with Maisson’s trichrome staining (blue positive area/total tissue area x 100%; veh *n* = 8, Bleo/vehicle *n* = 11, Bleo/BMS-986020 *n* = 12, Bleo/PIPE-791 *n* = 12, * *P* < 0.05, ** *P* < 0.01, n.s. non-significant, one-way ANOVA with Tukey’s, error bars SEM). **C** PIPE-791 reduces bleomycin induced lung hydroxyproline levels (veh *n* = 8, Bleo/vehicle *n* = 11, Bleo/BMS-986020 *n* = 12, Bleo/PIPE-791 *n* = 12, * *P* < 0.05, one-way ANOVA with Tukey’s, error bars SEM).

Upon histological examination by trichrome, we observed a significant induction of subpleural fibrosis in response to bleomycin insult, with the tissue collagen fraction (TCF) increasing from 7.09 to 10.77% (Fig. 8 A, *p* = 0.0479, vehicle/vehicle: 8 mice, 3 lung levels (rostral, medial, caudal)/mouse; bleo/vehicle: 11 mice, 3 lung levels/mouse). In mice treated with BMS-986020, we observed a 62% reduction in TCF (*p* = 0.2723, 12 mice, 3 lung levels/mouse). With PIPE-791 we observed a nearly full reduction with tissue collagen returning to vehicle/vehicle levels (Fig. 8B, *p* = 0.0017, 12 mice, 3 lung levels/mouse).

Hydroxyproline analysis also showed a strong induction of collagen after bleomycin insult, however mice administered BMS-986020 showed no reduction in hydroxyproline compared to bleomycin/vehicle mice. Additionally, the quantified hydroxyproline amounts were significantly higher than vehicle/vehicle mice. Mice dosed with PIPE-791, however, exhibited a 40% reduction in hydroxyproline compared to bleomycin/vehicle. Hydroxyproline levels in bleo/PIPE-791 treated mice were not significantly different from vehicle/vehicle treated mice (Fig. 8 C).

### PIPE-791 reduces macrophage activation and secretion of IL-1β

Macrophages undergo activation in response to inflammatory stimuli such as lipopolysaccharide (LPS) and bleomycin. Upon activation, these macrophages secrete inflammatory cytokines, e.g., IL-1β, and generating LPA via autotaxin, resulting in fibroblast activation, and thus implicated in fibrotic initiation and progression [29–31]. Because macrophages express LPAR1, we posited that inhibiting LPAR1 in an inflammatory, fibrosis-relevant context could lead to a reduction in macrophage-derived cytokine secretion.

**Fig. 9:**
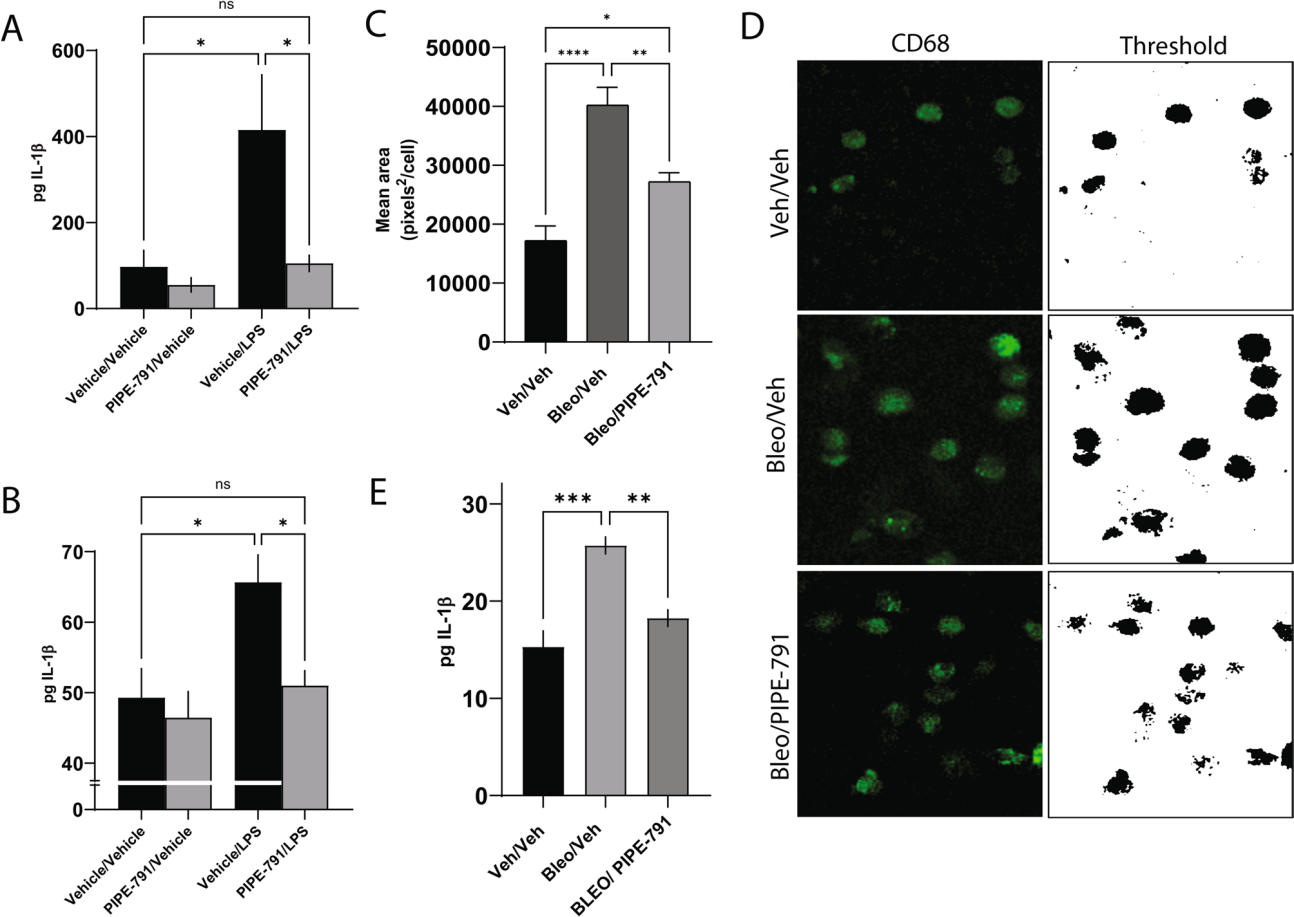
PIPE-791 regulates macrophage activation and release of IL-1β. **A** Primary human alveolar macrophages were treated with PIPE-791 then stimulated ex vivo with LPS. PIPE-791 significantly reduced IL-1 secretion into the culture media (*n* > 5, * *P* < 0.05, one-way ANOVA with Newman-Keuls, error bars SEM). **B** Alveolar macrophages were collected from mice orally dosed in vivo with 3 mg/kg PIPE-791 followed by ex vivo stimulation with LPS. Treatment with PIPE-791 reduced secreted IL-1β levels (*n* > 9, one-way ANOVA with Newman-Keuls, * *P* < 0.05, error bars SEM). **C** Bleomycin was administered intratracheally, then mice orally dosed with 3 mg/kg PIPE-791. BAL fluid was collected, separated into pellet and supernatant fractions, and analyzed. Cells were stained with the macrophage marker CD68, showing an increase in size (an indicator of activation) in response to bleomycin. Activation was quantified as the mean area of CD68 staining per cell. Bleomycin increased cell size which was reversed in mice dosed orally with PIPE-791 (*n* = 5, one-way ANOVA with Tukey’s, * *P* < 0.05, ** *P* < 0.01, **** *P* < 0.0001, error bars SEM). **D** Representative images of cells collected from BAL fluid in (**C**) stained with the macrophage marker CD68 (green, left column) showing an increase in size (an indicator of activation) in response to LPS stimulation. Thresholded images (right column). **E** Analysis of IL-1β in the BAL fluid showed a significant reduction in bleomycin induced IL-1β in mice dosed with PIPE-791 (*n* = 4, one-way ANOVA with Tukey’s, ** *P* < 0.01, *** *P* < 0.001, error bars SEM)

Given IL-1β’s role in promoting lung fibrosis, we asked whether activated alveolar macrophages could secrete IL-1β in vitro [32, 33]. In this experiment, primary human alveolar macrophages were stimulated ex vivo with LPS, resulting in a significant 4.3-fold increase in IL-1β in the culture media. This was inhibited to baseline levels (i.e. vehicle/vehicle) in the presence PIPE-791. No increase was observed with PIPE-791 alone (Fig. 9 A).

In a similar but separate experiment, mice were dosed orally with either vehicle or 3 mg/kg PIPE-791 before their alveolar macrophages were isolated 24 h after treatment, then stimulated ex vivo with LPS. A 1.3-fold increase in IL-1β was observed following LPS challenge in macrophages isolated from vehicle treated mice. In comparison, macrophages collected from PIPE-791 dosed mice showed IL-1β levels comparable to baseline (i.e. vehicle/vehicle) with and without ex vivo stimulation with LPS (Fig. 9B).

Based on these data, we believe that activated, LPAR1-expressing macrophages produce IL-1 and that PIPE-791 inhibits its secretion. These findings are congruent with work from other labs, showing that activated bone marrow derived macrophages similarly release IL-1β in an LPAR1 dependent manner [34].

### PIPE-791 reduces bleomycin induced IL-1β release in vivo

To further support our ex vivo macrophage findings, we used a therapeutic paradigm where mice were challenged in vivo with intratracheal instillation of bleomycin. Three days later, oral dosing with PIPE-791 (3 mg/kg, once a day) commenced, and BAL fluid was collected 11 days later. The fluid was centrifuged and supernatant and cell fractions analyzed.

In response to bleomycin, macrophages enter a proinflammatory state where they release cytokines and enlarge in size, due in part to increased protein synthesis and phagocytic activity [35]. Using CD68 as a cytosolic marker, macrophages were immunostained and analyzed for size. With bleomycin, we observed a significant 230% increase in cell size as measured by CD68 area. This was significantly reduced by 56% in mice given PIPE-791, suggesting a role for LPAR1 in macrophage activation (Fig. 9 C and D).

We also measured secreted IL-1β protein in the BAL supernatant and observed a 68% induction of IL-1β in the bleomycin treated group, followed by a significant 69.2% reduction in the presence of PIPE-791 (Fig. 9E).

## Discussion

Idiopathic pulmonary fibrosis is a chronic, progressive form of interstitial lung disease characterized by exaggerated amounts of extracellular matrix, alveolar damage, and tissue remodeling. Here, we have provided several lines of evidence in support of PIPE-791, a potent, orally bioavailable small molecule LPAR1 antagonist, as a treatment for lung fibrosis. PIPE-791 did not inhibit bile acid transporters or induce general and cholestatic toxicity in human hepatocyte sandwich cultures at pharmacologically relevant concentrations, thereby reducing the cholecystitic risk observed with early generation LPAR1 antagonists [12].

In vitro, we evaluated the activity of PIPE-791 using primary human lung fibroblasts whereby LPA addition induces COL1A1 production and promotes chemotaxis, two functions linked to LPAR1 activation and contribute to lung fibrosis. Inhibition was observed with both endpoints at low nanomolar potencies, in agreement with the recombinant cellular potency for PIPE-791 against LPAR1. In primary human lung fibroblast cultures, we also observed that PIPE-791 inhibited both TGFβ and LPA-induced fibroblast to myofibroblast transition as assessed by αSMA and COL1A1. In PCLS cultures or in the scar-in-a-jar model, where cells are at a high density, upregulation of fibrotic markers by TGFβ or LPA suggest that the observed increase are mechanistically distinct pathways [36]. Our data in a more dispersed setting adds to these observations and suggests a point during the transformation into myofibroblasts, where fibroblasts activated independently by either insult is sensitive to LPAR1 antagonism. Interestingly, in lung fibroblasts, TGFβ1 induces IL-6 expression, which can lead to the induction of autotaxin and increased LPA concentrations [13, 14, 37]. While further work is warranted, this suggests a stage during fibroblast activation where an LPAR1 antagonist may be more generally efficacious in preventing myofibroblast differentiation.

Human precision cut lung slices have become important as an ex vivo model for better understanding lung fibrosis in a human context [38]. Although they do not capture all the complexities of the diseased condition, they offer an opportunity to assess the efficacy of a compound for the treatment of fibrosis in a human setting. Having shown elevated LPAR1 expression in IPF tissue sections by autoradiography, we obtained cryopreserved PCLS tissue from normal donors and donors with pulmonary fibrosis. Treatment of diseased PCLS with PIPE-791 significantly reduced *COL1A1*, *COL3A1*, *SERPINE1*, and *TIMP1*. We also tested two other anti-fibrotic mechanisms in PCLS using two clinical-stage small molecules, an αvβ1/6 inhibitor (bexotegrast, PLN-74809) and a PDE4B inhibitor (nerandomilast, BI 1015550). In both cases we observed some downregulation of fibrotic markers, but some markers failed to reach statistical significance, suggesting LPAR1 as a mechanism may be superior. Although these would need to be tested empirically, it would also stand to reason that PIPE-791 in combination with another mechanism, such as a PDE4B inhibitor, would have the capacity to demonstrate additive efficacy. Nonetheless, these data show that in diseased human PCLS cultures, PIPE-791 reduces the expression of known fibrosis markers.

Biomarkers have recently become of enormous clinical interest in pulmonary fibrosis for their utility in assessing disease state and compound efficacy. In a collaboration with Nordic Biosciences and Fraunhofer ITEM using fresh, non-cryopreserved IPF PCLS tissue, we observed significant reduction of two secreted biomarkers, C3M and ProC6, following incubation with PIPE-791. While these data further support that PIPE-791 inhibits fibrogenesis and tissue remodeling in diseased human tissue, they also provide valuable insight into which biomarkers should be monitored in future clinical studies.

Currently, there are no approved LPAR1 receptor antagonists to treat IPF. Within this class, BMS-986278 is the furthest in development, having shown improvement in IPF patients in a Phase II setting. Results reveal that 60 mg, given twice daily, is required for clinical benefit. To identify a compound with improved properties, allowing for convenient once daily dosing, we focused our attention on in vivo properties that would result in sustained receptor coverage. A combination of factors including high intrinsic LPAR1 potency, decreased plasma protein binding, good oral bioavailability, and improved metabolic stability of PIPE-791 converge to afford full coverage of the LPAR1 receptor in excess of 24 h after a single, low oral dose. This was confirmed by functional measurements, using LPA-induced histamine release, and by imaging, using competitive displacement with a radiolabeled LPAR1 antagonist in the lung. In both settings, we observe dose dependent effects with full target engagement from 3 to 24 h following a single oral dose of 3 mg/kg. With repeat dosing, a lower dose of 0.3 mg/kg, once daily, provided full target engagement. To the best of our knowledge, from an in vivo perspective, these results suggest PIPE-791 represents the most potent and longest lasting small molecule LPAR1 receptor antagonist discovered to date.

We confirmed efficacy and dose translation in mouse using two methods of bleomycin delivery, oropharyngeal and subcutaneous, to induce lung fibrosis. Oropharyngeal bleomycin results in a rapid increase in lung collagen as well as an increase in LPAR1 measured by autoradiography. PIPE-791, given prophylactically, dose-dependently reduced lung collagen with a maximal inhibition of 40% at a dose level of 0.3 mg/kg. This level of collagen reduction is consistent with that observed with other LPAR1 antagonists and in LPAR1 KO mice [4, 5]. Maximal reduction of other BAL fluid fibrosis biomarkers requires a slightly higher dose of 3 mg/kg. Based on these results, we elected to evaluate the higher dose of 3 mg/kg in the more chronic subcutaneous bleomycin lung fibrosis model. When administered therapeutically, this dose level reduced lung collagen to control/non-bleomycin values. On the other hand, BMS-986020, another LPAR1 antagonist that had shown promising efficacy in IPF patients, given at 30 mg/kg, twice daily, only resulted in a modest reduction by histology [39]. These results confirm that PIPE-791 is effective in reducing lung fibrosis and highlights the in vivo potency of this molecule.

We were also intrigued by the role of alveolar macrophages in lung fibrosis and were able to demonstrate that in vivo treatment with bleomycin induced an increase in macrophage size and IL-1β release and that both were inhibited with PIPE-791. As such, these data suggest a role for LPA and LPAR1 in macrophage activation during IPF [40, 41]. We suggest that LPAR1 activation contributes to macrophage activation and cytokine release, leading to the transformation of lung fibroblasts and fibrosis progression [32, 33]. It is possible that measuring IL-1β in BALF could be used to assess LPAR1 target engagement. Of note, IL-1β can also be measured from patient sputum, supporting its utility as a potential biomarker [42].

PIPE-791 has been found to be safe and well-tolerated in a Phase I clinical trial https://clinicaltrials.gov/study/NCT05983939 (Registration date: 2023-07-19). PIPE-791 is also currently in a PET study to better understand receptor occupancy in human brain and lung (https://clinicaltrials.gov/study/NCT06683612). Together with the data presented here, as well as previous data showing LPAR1 involvement in IPF, we believe that there is a strong impetus for the development of PIPE-791 as a treatment for lung fibrosis.

## Methods

### Calcium flux

Plasmids containing human LPAR1-6 were obtained from Origene (Rockville, MD) and maxiprepped at Eton Biosciences (San Diego, CA). Transfection mix was prepared by adding Fugene 6 transfection mix to serum free media and 0.1 µg plasmid. After a 20 m incubation, HEK293T cells (ATCC, Manassas VA) were lifted with Accutase and added to the Fugene mix at a final concentration of 0.5 × 10^4^ cells/well. Cells were then plated on black clear bottom 96-well plates and incubated overnight in a 37 °C, 5% CO_2_ incubator. After 24 h, varying concentrations of PIPE-791 were added and cells were returned to the incubated for 3 h. During the last hour of incubation, media was removed and replaced with Fluo-4 NW calcium assay dye prepared as per manufacturer’s recommendations in assay buffer. Agonist plates containing LPA were prepared for individual receptor subytpes using their respective EC_80_. Calcium mobilization was measured as per manufacturer’s directions on a Flexstation 3 (Molecular Devices, San Jose, CA).

### Cholestatic drug-induced liver injury (c-DILI) assessment

This study was conducted at BioIVT (Durham, CO). Sandwich-cultured human hepatocytes (SCHH) were prepared on BioCoat 96-well Cell Culture Plate from Corning (Cat # 354407) using Transporter Certified^™^ cryopreserved hepatocytes from BioIVT (Cat # M00995-TCERT) and maintained in freshly prepared QualGro^™^ Hepatocyte Culture Medium. On day 4 of culture, SCHH were treated with QualGro^™^ Sensitization Medium (to assess cholestatic hepatotoxcity) or QualGro^™^ Human Culture Medium (to assess general hepatotoxicity) containing solvent control DMSO (0.1% v/v), Cyclosporine (10 µM, negative control), Troglitazone (75 µM, positive control), Imatinib, (40 µM, positive control), or PIPE-791 (1, 3, 10, and 30 µM). Following 24 h of exposure, the culture plates were harvested for adenosine triphosphate (ATP) and lactate dehydrogenase (LDH) analysis. Cellular ATP content was determined using the CellTiter-Glo^®^ Assay Kit and LDH leakage into the culture media was determined using the CytoTox-ONE^®^ Assay Kit, both obtained from Promega, per manufacturer’s instructions.

### Human lung fibroblast chemotaxis and COL1A1 induction

Chemotaxis assay: Primary human lung fibroblasts were obtained from ATCC (Cat # PCS-201-020) and cultured in fibroblast media as per ATCC directions. Fibroblasts were serum starved overnight in basal fibroblast media. Cells were then lifted using CellStripper, centrifuged at 500 x g for 10 min, then resuspended in basal media. Cells in suspension were incubated in varying concentrations of PIPE-791 for 6 h then transferred to a chemotaxis chamber and incubated for 6 h. The receiving chamber contained 1 µM LPA. Chemotaxis was quantified as per Cell Biolabs, Inc instructions on a Flexstation 3 (Molecular Devices).

COL1A1 assay: Primary human lung fibroblasts were plated in a 96-well plate and allowed to adhere. Cells were serum starved for 4 h then PIPE-791 or BMS-986278 was added at varying concentrations and cells incubated overnight. The next day, cells were treated with 1 µM LPA and incubated for 8 h. After incubation in LPA, cells were immunostained for COL1A1. Cells were fixed in formalin for 10 min, washed 3 x with PBS, incubated in collagen 1A1 antibody (1:250 in blocking buffer, 0.1% Triton X-100 in PBS, 2 h), and washed 3 x with PBS. Secondary antibody solution (1:500) and Hoechst (1:2000) were added for 1 h, washed 3 x in PBS then imaged on a Nikon A1R confocal microscope at 10x magnification except human lung fibroblasts which were imaged at 4x. Collagen area was quantified with ImageJ and normalized to Hoechst per image.

SMA and COL1A1 stimulation with TGFβ or LPA: Primary human lung fibroblasts were plated and treated with PIPE-791 in a similar manner to the COL1A1 assay with the following modifications: Cells were plated on poly-D-lysine coated plates. TGFβ1 was used at 5 ng/mL. In addition to COL1A1 immunostaining, cells were co-immunostained with aSMA followed by a goat anti-rabbit Alexa647 secondary antibody.

### Human tissue autoradiography

Fresh frozen human lung samples were obtained from BioIVT and kept at −80 °C (Additional File 1). Lung tissue blocks were sectioned at 20 μm with a Thermo Shandon FSE cryostat and mounted on Superfrost Plus^®^ slides (VWR, Radnor, PA). Sections were dried and slides stored at −80 °C until the day of the experiment.

Sections were dried for 1 h at room temperature and incubated in binding buffer (HEPES 50 mM, NaCl 100 nM, EDTA 2 mM, pH 7.4) for 15 min at room temperature in a humidified chamber. Then binding buffer was removed carefully and sections were covered with 0.3 ml of [^3^H]-PIPE-497 (20 nM, specific activity 35.8 Ci/mmol, Vitrax, Placentia, CA, USA) in binding buffer for 90 min at room temperature in a humidified chamber. Non-specific binding (NSB) was evaluated using adjacent sections incubated in binding buffer containing the same amount of radiotracer and 15 µM of cold OPC-497. Sections were rinsed 2 × 1 min in washing buffer (Tris 50 mM, NaCl 154 mM, Tween 20 0.05%, pH 7.4) at room temperature followed by two 15 s dips in 4 °C distilled water. Slides were dried with cold air flow and exposed to a tritium sensitive storage phosphor screen (Cytiva, BAS-IP TR 2025 E, Marlborough, MA, USA) for 10–21 days. The imaging plate was scanned on a Storm 860 (Molecular Dynamics, Sunnyvale, CA) scanner with 50 μm resolution.

For analysis the total amount of radioligand bound to each section of interest was measured using ImageJ imaging software (ver 1.53t, NIH, USA W. Rasband). Two sections per patient were analysed and relative optical density determined from two 300 μm square regions of interest for each section. Replicate measurement were plotted using Prism (v10.4.0, GraphPad, San Diego, USA) and bar graphs generated for each patient.

### Human precision cut lung slices

Precision cut lung slices were obtained from Anabios (San Diego, CA) and collected in accordance with their ethics policy (https://anabios.com/ethics-statement/). Donor demographics are available in Additional File 1. Slices were thawed as per vendor instructions. Briefly, PCLS were thawed at 37 °C then transferred to media consisting of DMEM/F12, Antibiotic-Antimytotic, and Penicillin-Streptomycin. PCLS were gently rinsed then transferred to a larger dish. A small tissue sample was excised with a scalpel for immunohistochemistry and qPCR characterization. Slices were cultured for 24 h in culture media, then replaced with media plus vehicle, BI 1015550, PLN-74809, or PIPE-791 dissolved in DMSO. Final concentration of DMSO was 0.1%. Media and compound were replaced every two days for a total of 6 days. At the end of the experiment, slices were collected and snap frozen. *Quantitative PCR*: RNA was extracted using RNEasy columns. RNA was reverse transcribed using qScript XLT per manufacturer’s instructions. Genes of interest were assessed by qPCR using Perfecta SYBR Green on a StepOne Plus Thermocycler (ABI). Parameters were as follows: 95 °C 30 s, then 45 cycles of 95 °C for 5 s and 60 °C for 30 s.

PCLS cultures for secreted biomarker analysis were performed at Fraunhofer ITEM (Hanover, Germany) using tissue from pathologist qualified, pulmonary fibrosis patients. Tissue from individual donors and 2 lung regions were collected in accordance with policies approved by the Hanover Medical School ethics committee. Only fibrotic tissue was used for experiments. Region 1 consisted of subpleural tissue and Region 2 of central tissue. For each, lung lobes were filled with low-melting agarose then sectioned at 300 μm on a Krumdiek tissue slicer as described in Hesse et al., 2022. Slices were incubated in DMEM containing penicillin and streptomycin, two slices per region were used. Slices are left overnight then incubated in vehicle (DMSO), nintedanib (300 nM), or 0.03, 0.3 or 3 µM PIPE-791 for 48 h. Supernatant was collected and shipped to Nordic Bioscience (Herlev, Denmark) for C3M, Pro-C6 and Pro-C3 analysis. Donor information can be found in Additional File 1.

### Animal care

All animal procedures were approved by the Contineum Therapeutics Institutional Animal Care and Use Committee. Mice and rats (Charles River or Envigo) were acclimated for approximately at least 1 week. Rodents were multi-housed in Innorack IVC racks (Innovive, San Diego, CA) with access to water and standard rodent chow *ad libitum*. Animals were subject to a 12 h light – 12 h dark cycle.

### Mouse lung receptor occupancy

Female C57BL6/N (Envigo) mice were dosed with vehicle or PIPE-791 by oral gavage. [^3^H]-PIPE-497 was diluted to 9.8 µCi/mL in saline and administered via IV injection to the lateral tail vein at a dose volume of 5 mL/kg. Five minutes following radioligand administration, mice were euthanized by decapitation, trunk blood collected into K_3_EDTA tubes and stored on wet ice, each lung rapidly dissected, weighed and placed into a 5 mL polypropylene tube. Tissues were diluted with a 10x volume of ice-cold binding buffer (50 mM HEPES, 100 mM NaCl_2_, 2 mM EDTA). Lungs were then homogenized (25,000 rpm, 7 s). 300 µL of homogenate was filtered over Whatman GF/B filters (GE Life Sciences, Marlborough, MA) which had been pre-wetted with 0.5% polyethyleneimine prior to loading onto the Hoefer vacuum manifold. Filters were washed once by applying 3 mL ice-cold wash buffer (50 mM Tris, 154 mM NaCl, 0.05% Tween 20) to the manifold for a total of 2 washes. Remaining homogenate was stored on ice and transferred to −80 °C for storage until analysis of PIPE-791 by LC-MS/MS. Washed filters were removed from the manifold and air dried. Following completion of filtration of all samples, uncapped scintillation vials were placed into an oven (~ 40–50 °C) for ~ 30 min to ensure complete drying. Five (5) mL Ultima Gold F scintillation fluid was added to each tube. Tubes were capped, gently agitated, and dark equilibrated for 30 min then read on a Beckman LS6500 liquid scintillation counter for tritium disntegrations per minute measurements. After all samples were collected, dissected, and filtered, the previously collected blood in K_3_EDTA tubes was centrifuged (1450 x g, 10 min, 4 °C) to separate plasma. Plasma was aliquoted into 96-well polypropylene plates and stored at −80 °C until analysis for PIPE-791 by LC-MS/MS.

### In vivo mouse histamine release

Female CD-1 mice (Envigo) were allowed to acclimate for ~ 1 week. Animals were subject to a 12 h light – 12 h dark cycle. Mice were multi-housed in Innorack IVC mouse racks (Innovive, San Diego, CA) with access to water and standard rodent chow *ad libitum*. Mice were dosed by oral gavage with a single dose of vehicle or PIPE-791 or with 4 repeated once daily doses. Three hours or 24 h after a single dose or after the last of 4 repeated administrations, 150 µL FAF-PBS/BSA (Control) or LPA (300 µg in 150 µL) was administered IV via the lateral tail vein and mice were immediately placed into an isoflurane chamber. Two minutes later, mice were euthanized by decapitation and trunk blood collected into K_3_EDTA tubes and stored on wet ice until processing to plasma (1450 *x g*, 10 min, 4 °C). Plasma was aliquoted into two 96-well polypropylene plates and transferred to −80 °C for storage until analysis for histamine by EIA.

### Intratracheal bleomycin mouse model

Female C57BL6/N mice 8–10 weeks old were obtained from Charles River. On Day − 1, vehicle (1% HPMC/0.1% Tween 80) or PIPE-791 (0.003, 0.03, 0.3, or 3 mg/kg) was administered by oral gavage (10 mL/kg) 23 h prior to oropharyngeal instillation of sterile saline or bleomycin on Day 0 and then daily throughout the duration of the experiment. Bleomycin was administered by oropharyngeal instillation 23 h after first dose (Day 0). On the day of instillation, bleomycin was prepared by diluting a 15 unit (U) vial to 1.5 U/mL with sterile saline. Mice were lightly anesthetized with isoflurane (5% in 100% O_2_) and administered sterile saline or bleomycin (3.0 U/kg) via oropharyngeal instillation at a dose volume of 2 mL/kg (2 µL/g). Mice were returned to their cages and monitored daily for the duration of each experiment. Vehicle (1% HPMC/0.1% Tween 80) or PIPE-791 (0.003, 0.03, 0.3, or 3 mg/kg) were delivered once daily by oral gavage over the course of the study. Fourteen days after bleomycin instillation, mice were euthanized via isoflurane anaesthesia, cardiac blood collected into K_3_EDTA tubes and stored on wet ice, and BALF and lungs were isolated for subsequent analysis of biochemical and histopathological indices of lung injury, inflammation, and fibrosis. To do this, mice were intubated with a 20-gauge luer stub adapter connected to a 3-way stopcock fitted with two 3 mL syringes (the delivery syringe was pre-filled with 1.5 mL phosphate buffered saline (PBS), and the receiving syringe was empty). Lungs were lavaged with PBS (2 × 0.75 mL) to obtain BALF. BALF was placed on wet ice until centrifugation to separate from cells. Lungs were dissected and placed into 1 mL 6 M HCl for collagen analysis using the Quickzyme total collagen assay kit according to manufacturer’s instructions. At the end of the experimental collection, BALF was centrifuged (300 x g, 10 min, 4 °C) to isolate BALF from the cell pellet. The cell pellet was discarded and the BALF aliquoted and stored at −80 °C for biomarker analysis. For the analysis, *n* = 8 biological replicates were analyzed in singlicate for various biomarkers (total protein, TGFβ1, TIMP-1, procollagen) according to manufacturer’s recommended instructions.

### Mouse lung tissue autoradiography binding

Female C57BL6/N (Envigo) mice 8–10 weeks old were obtained from Charles River. Bleomycin (3.0 U/kg) or sterile saline were administered by oropharyngeal instillation under light isoflurane anesthesia (5% in 100% O_2_) at a volume of 2 mL/kg (2 µL/g). Mice (*n* = 3–4/group) were returned to their cages and monitored daily for the duration of each experiment. Three days post bleomycin injection, mice were deeply anesthetized with isoflurane and lungs were lavaged with PBS (2 × 0.75 mL) as described previously and injected with 0.75 ml (M1 embedding medium, Epredia, Kalamazoo, MI USA). Whole lungs were dissected, embedded in OCT Tissue Tek compound (Sakura Finetek, Torrance, CA, USA) and immediately frozen before sectioning. Lung were sectioned at 20 μm with a Thermo Shandon FSE cryostat and mounted on Superfrost Plus^®^ slides (VWR, Radnor, PA). Sections were dried and slides stored at −80 °C until the day of the experiment.

Autoradiography binding was performed in the same manner as human lung samples using 15 nM of [^3^H]-PIPE-497. Analysis was done on whole lung sections. Three sections per animal were used to generate mean oprtical density.

### Systemic bleomycin mouse model

This study was conducted by Inotiv (West Lafayette, IN). Forty-four female 9 week old C57Bl/6 mice (Jackson Labs) were provided standard chow (Envigo, Cat #: 8940), housed under standard conditions, and allowed to acclimate for > 3 days prior to enrollment. Prior to start, mice were weighed and placed into balanced enrollment groups. Four days prior to start, mice were anesthetized, dorsal fur shaved and removed with depilatory agent, and two circles (~ 1.5 cm diameter) heat-branded on their dorsal abdomen. Fur was removed as needed for the balance of the study to ensure consistent location of s.c. injections. Beginning on day 0 and through day 26, mice were anesthetized with isoflurane and administered 0.9% NaCl or bleomycin injections (100 µl/injection, s.c.) into each of the circles (2 injections/mouse/d) on a 5 day on/2 day off paradigm. Mice were administered 0.9% NaCl (vehicle) or bleomycin (0.1 U) with bleomycin doses split evenly between the two circles (i.e., 0.05 U/circle). Compounds were then orally administered: PIPE-791 (3 mg/kg, QD) or BMS-986020 (30 mg/kg, BID) from day 0 to 28. Weight measures and health observations were made daily. On day 27, mice were dosed to enable trough blood/plasma collection on day 28, which was collected via retro-orbital approach under isoflurane anesthesia. Following blood collection, mice were administered vehicle or compound on a timetable as appropriate to enable a peak blood collection 2 h postdose. At the time of sacrifice, mice were anesthetized with pentobarbital, a tracheotomy performed and interfaced with a positive pressure ventilator providing 100% O_2_. The mouse’s chest was then opened, and a needle interfaced with an infusion pump introduced to the right ventricle. The inferior vena cava and aorta was sectioned, and the infusion pump engaged to perfuse the pulmonary vasculature with 0.9% NaCl to remove all blood from the lung. The lungs were then harvested, immediately immersed in ice-cold 0.9% NaCl, gross dissected and subjected to morphological analyses. Tissue samples will be processed for biochemical and/or histological analyses as described below. Masson’s Trichrome Blue stained slides were subjected to histological analyses using color segmentation analysis for lung fibrosis. Frozen lung samples underwent hydroxyproline analysis as described below. *Lung tissue processing –* following exsanguination and flushing of the pulmonary circulation to clear blood, the lungs were removed, placed in ice-cold 0.9% NaCl and processed on wet ice. Extraneous tissue was removed and whole lung weight recorded. The right and left main bronchi were separated with the right bronchus ligated. The right bronchus was sectioned distal to the ligature with left and right lobes prepared for histological and biochemical analysis, respectively. *Histological Sample*: Left lungs from all animals were 10% NBF-fixed. After removal of the right lung, a length of PE-50 interfaced with a 1 mL syringe containing 10% NBF was inserted into the left bronchus via the trachea. The tubing was secured with tied suture and the lung gently inflated with 10% NBF until fully distended, but no fluid leaking from the lung. The tubing was removed, the bronchus ligated, and the inflated lung immersed in 10% NBF for 48 h. After 48 h of fixation, the suture was removed, and the lung transferred to 70% EtOH for storage and until processed for histological analysis. *Hydroxyproline*: The right lung was weighed, then placed flash frozen on liquid N_2_ and stored at −80 °C until powdered on liquid nitrogen by mortar and pestle. Approximately 30–40 mg of powdered lung was used for hydroxyproline analysis.

### IL-1β measurements in alveolar macrophages

Human macrophages were cultured in Human Alveolar Macrophage Media per manufacturer’s instructions. Cell were plated at 150,000 cell/well and incubated in the presence or absence of PIPE-791. Cells were then co-stimulated with 500 ng/mL LPS for 4 h. Plates were spun down and supernatant collected for analysis using a human IL-1β ELISA kit per manufacturer’s directions.

To collect mouse alveolar macrophages, CD-1 mice (Charles River) were orally dosed with vehicle or 3 mg/kg PIPE-791 24 h prior to collection. 1.5 mL DPBS was injected into lungs and BAL fluid aspirated. Cells were plated in at 40,000 cells/well and stimulated first with 500 ng/mL LPS for 4 h then and 1 µM LPA for an additional hour at 37 °C. Plates were spun down and supernatant analyzed for IL-1β by ELISA.

To collect alveolar macrophages from bleomycin treated mice, CD-1 mice (Charles River) were dosed intratracheally with bleomycin (3 units/kg) then dosed orally with vehicle or PIPE-791 (3 mg/kg, once a day) commencing 3 d later. Mice were dosed until day 14 at which point BAL fluid was collected. BAL was centrifuged and cells were transferred to culture plates and incubated overnight at 37 °C. Plates were spun down and supernatant and cell pellet analysed separately. Supernatant was analyzed for IL-1β as described for mouse above. The cell pellet for each condition was plated at 3 × 10^3^ cells/well in black clear bottom 96-well plates. After settling, cells were fixed in formalin and immunostained against CD68 and detected with a donkey anti-rat Alexa 488 conjugated antibody. Image acquisition and analysis was performed using a Nikon A1R confocal microscope and NIS Elements software.

### Bioanalysis of mouse or rat plasma for PIPE-791

Calibration standards and QC solutions of PIPE-791 were prepared in solvent and spiked into naive mouse plasma or lung homogenate matrixes to generate calibration curves and quality controls. Plasma and lung homogenate samples were thawed and further diluted as necessary with naïve mouse plasma or naïve lung homogenate matrix. For PIPE-791 drug analysis, protein precipitation was performed on all samples/standards/quality controls using 1:4 v/v ratio of acetonitrile quench solution containing the internal standard IS3064, the stable isotope labelled test article (PIPE-791-d_9_; Contineum Therapeutics, San Diego, CA). Next, samples were centrifuged (4000 x g, 10 min, 4 °C) and supernatants injected for LC-MS/MS analysis of PIPE-791 (Sciex 4000 QTrap, Framingham, MA, USA). Plasma drug concentrations were calculated by comparison of peak area ratios of analyte/IS to the prepared calibration standards using Sciex Analyst software. The protein binding profile of PIPE-791 in mouse plasma was assessed by the equilibrium dialysis method and used to calculate plasma free drug concentrations.

### Reagents

Vendor and catalog numbers can be found in the supplementary material (Reagent List).

### Statistics

Graphs were generated with GraphPad Prism using tests as described in figure legends.

## Supplementary Information

Supplementary Material 1.

Supplementary Material 2.

Supplementary Material 3.

## Data Availability

The data supporting the findings of this manuscript are available from the corresponding author upon reasonable request, however, some of the datasets generated may not be publicly available due to commercial confidentiality and proprietary restrictions as they contain intellectual property owned by Contineum Therapeutics. Requests for data will be evaluated by Contineum Therapeutics to ensure compliance with applicable confidentiality agreements, intellectual property protections, and regulatory requirements. Data sharing will also be subject to a data use agreement. Due to commercial confidentiality and ongoing patent applications, the chemical structure of the novel proprietary compound used in this study cannot be publicly disclosed. Reasonable requests by researchers for access for internal, academic, non-commercial research purposes will be considered on a case-by-case basis by the corresponding author after submission of a formal request and will require the execution of a Material Transfer Agreement (MTA) with Contineum Therapeutics due to confidentiality and intellectual property considerations and in order to track use of the compound for further research.

## References

[1] Raghu G, Remy-Jardin M, Richeldi L, Thomson CC, Inoue Y, Johkoh T, Kreuter M, Lynch DA, Maher TM, Martinez FJ, et al. Idiopathic pulmonary fibrosis (an Update) and progressive pulmonary fibrosis in adults: an official ATS/ERS/JRS/ALAT clinical practice guideline. Am J Respir Crit Care Med. 2022;205:e18–47. 35486072 10.1164/rccm.202202-0399ST PMC9851481

[2] Selman M, Pardo A. The leading role of epithelial cells in the pathogenesis of idiopathic pulmonary fibrosis. Cell Signal. 2020;66:109482. 31760172 10.1016/j.cellsig.2019.109482

[3] Glass DS, Grossfeld D, Renna HA, Agarwala P, Spiegler P, DeLeon J, Reiss AB. Idiopathic pulmonary fibrosis: current and future treatment. Clin Respir J. 2022;16:84–96. 35001525 10.1111/crj.13466 PMC9060042

[4] Swaney JS, Chapman C, Correa LD, Stebbins KJ, Bundey RA, Prodanovich PC, Fagan P, Baccei CS, Santini AM, Hutchinson JH, et al. A novel, orally active LPA(1) receptor antagonist inhibits lung fibrosis in the mouse bleomycin model. Br J Pharmacol. 2010;160:1699–713. 20649573 10.1111/j.1476-5381.2010.00828.x PMC2936842

[5] Tager AM, LaCamera P, Shea BS, Campanella GS, Selman M, Zhao Z, Polosukhin V, Wain J, Karimi-Shah BA, Kim ND, et al. The lysophosphatidic acid receptor LPA1 links pulmonary fibrosis to lung injury by mediating fibroblast recruitment and vascular leak. Nat Med. 2008;14:45–54. 18066075 10.1038/nm1685

[6] Volkmann ER, Denton CP, Kolb M, Wijsenbeek-Lourens MS, Emson C, Hudson K, Amatucci AJ, Distler O, Allanore Y, Khanna D. Lysophosphatidic acid receptor 1 inhibition: a potential treatment target for pulmonary fibrosis. Eur Respir Rev. 2024;33(172). 10.1183/16000617.0015-2024 PMC11262619 39009409

[7] Choi JW, Herr DR, Noguchi K, Yung YC, Lee CW, Mutoh T, Lin ME, Teo ST, Park KE, Mosley AN, Chun J. LPA receptors: subtypes and biological actions. Annu Rev Pharmacol Toxicol. 2010;50:157–86. 20055701 10.1146/annurev.pharmtox.010909.105753

[8] Fransson J, Gomez-Conde AI, Romero-Imbroda J, Fernandez O, Leyva L, de Fonseca FR, Chun J, Louapre C, Van-Evercooren AB, Zujovic V, et al. Activation of macrophages by lysophosphatidic acid through the lysophosphatidic acid receptor 1 as a novel mechanism in multiple sclerosis pathogenesis. Mol Neurobiol. 2021;58:470–82. 32974731 10.1007/s12035-020-02130-x

[9] Jiang S, Yang H, Li M. Emerging roles of lysophosphatidic acid in macrophages and inflammatory diseases. Int J Mol Sci. 2023;24(15). 10.3390/ijms241512524 PMC10419859 37569902

[10] Neighbors M, Li Q, Zhu SJ, Liu J, Wong WR, Jia G, Sandoval W, Tew GW. Bioactive lipid lysophosphatidic acid species are associated with disease progression in idiopathic pulmonary fibrosis. J Lipid Res. 2023;64:100375. 37075981 10.1016/j.jlr.2023.100375 PMC10205439

[11] Poon MM, Lorrain KI, Stebbins KJ, Edu GC, Broadhead AR, Lorenzana AO, Paulson BE, Baccei CS, Roppe JR, Schrader TO, et al. Discovery of a brain penetrant small molecule antagonist targeting LPA1 receptors to reduce neuroinflammation and promote remyelination in multiple sclerosis. Sci Rep. 2024;14:10573. 38719983 10.1038/s41598-024-61369-9 PMC11079064

[12] Gill MW, Murphy BJ, Cheng PTW, Sivaraman L, Davis M, Lehman-McKeeman L. Mechanism of hepatobiliary toxicity of the LPA(1) antagonist BMS-986020 developed to treat idiopathic pulmonary fibrosis: contrasts with BMS-986234 and BMS-986278. Toxicol Appl Pharmacol. 2022;438:115885. 35090952 10.1016/j.taap.2022.115885

[13] Eickelberg O, Kohler E, Reichenberger F, Bertschin S, Woodtli T, Erne P, Perruchoud AP, Roth M. Extracellular matrix deposition by primary human lung fibroblasts in response to TGF-beta1 and TGF-beta3. Am J Physiol. 1999;276:L814–824. 10330038 10.1152/ajplung.1999.276.5.L814

[14] Castelino FV, Bain G, Pace VA, Black KE, George L, Probst CK, Goulet L, Lafyatis R, Tager AM. An autotaxin/lysophosphatidic Acid/Interleukin-6 amplification loop drives scleroderma fibrosis. Arthritis Rheumatol. 2016;68:2964–74. 27390295 10.1002/art.39797 PMC5125861

[15] Morse C, Tabib T, Sembrat J, Buschur KL, Bittar HT, Valenzi E, Jiang Y, Kass DJ, Gibson K, Chen W, et al. Proliferating SPP1/MERTK-expressing macrophages in idiopathic pulmonary fibrosis. Eur Respir J. 2019;54(2). 10.1183/13993003.02441-2018 PMC8025672 31221805

[16] Donnelly D, Kim J, Tran T, Cheng P, Corte J, Fang T, Murphy B, Shorts A, Kalinowski S, Cao K, et al. <strong > Design, synthesis and discovery of a second-generation PET ligand for lysophosphatidic acid receptor 1 (LPA1) to measure target engagement of LPA1 antagonists in lung tissues</strong >. J Nucl Med. 2025;66:251230–251230.

[17] Koziol-White C, Gebski E, Cao G, Panettieri RA Jr. Precision cut lung slices: an integrated ex vivo model for studying lung physiology, pharmacology, disease pathogenesis and drug discovery. Respir Res. 2024;25:231. 38824592 10.1186/s12931-024-02855-6 PMC11144351

[18] Machahua C, Marti TM, Dorn P, Funke-Chambour M. Fibrosis in PCLS: comparing TGF-beta and fibrotic cocktail. Respir Res. 2025;26:44. 39875887 10.1186/s12931-025-03110-2 PMC11776118

[19] Decaris ML, Schaub JR, Chen C, Cha J, Lee GG, Rexhepaj M, Ho SS, Rao V, Marlow MM, Kotak P, et al. Dual Inhibition of alpha(v)beta(6) and alpha(v)beta(1) reduces fibrogenesis in lung tissue explants from patients with IPF. Respir Res. 2021;22:265. 34666752 10.1186/s12931-021-01863-0 PMC8524858

[20] Herrmann FE, Hesslinger C, Wollin L, Nickolaus P. BI 1015550 is a PDE4B inhibitor and a clinical drug candidate for the oral treatment of idiopathic pulmonary fibrosis. Front Pharmacol. 2022;13:838449. 35517783 10.3389/fphar.2022.838449 PMC9065678

[21] Hesse C, Beneke V, Konzok S, Diefenbach C, Bulow Sand JM, Ronnow SR, Karsdal MA, Jonigk D, Sewald K, Braun A, et al. Nintedanib modulates type III collagen turnover in viable precision-cut lung slices from bleomycin-treated rats and patients with pulmonary fibrosis. Respir Res. 2022;23:201. 35927669 10.1186/s12931-022-02116-4 PMC9351157

[22] Jenkins G, Maher TM, Cottin V, Nishioka Y, Noth I, White ES, Ittrich C, Diefenbach C, Rohr KB, Stowasser S, Selman M. Effect of nintedanib on blood biomarkers in patients with IPF in the Inmark trial. Eur Respir J. 2019;54(Suppl 63):PA2254.

[23] Kobayashi Y, Uneuchi F, Naruse T, Matsuda D, Okumura-Kitajima L, Kajiyama H, Wada R, Yonemoto Y, Nakano K, Toki H, et al. Lead generation from N-[benzyl(4-phenylbutyl)carbamoyl]amino acid as a novel LPA(1) antagonist for the treatment of systemic sclerosis. Eur J Med Chem. 2023;260:115749. 37639822 10.1016/j.ejmech.2023.115749

[24] Qian Y, Hamilton M, Sidduri A, Gabriel S, Ren Y, Peng R, Kondru R, Narayanan A, Truitt T, Hamid R, et al. Discovery of highly selective and orally active lysophosphatidic acid receptor-1 antagonists with potent activity on human lung fibroblasts. J Med Chem. 2012;55:7920–39. 22894757 10.1021/jm301022v

[25] Swaney JS, Chapman C, Correa LD, Stebbins KJ, Broadhead AR, Bain G, Santini AM, Darlington J, King CD, Baccei CS, et al. Pharmacokinetic and pharmacodynamic characterization of an oral lysophosphatidic acid type 1 receptor-selective antagonist. J Pharmacol Exp Ther. 2011;336:693–700. 21159750 10.1124/jpet.110.175901

[26] Hashimoto T, Ohata H, Honda K. Lysophosphatidic acid (LPA) induces plasma exudation and Histamine release in mice via LPA receptors. J Pharmacol Sci. 2006;100:82–7. 16404130 10.1254/jphs.fpj05030x

[27] Harrison JH Jr., Lazo JS. High dose continuous infusion of bleomycin in mice: a new model for drug-induced pulmonary fibrosis. J Pharmacol Exp Ther. 1987;243:1185–94. 2447265

[28] Leach HG, Chrobak I, Han R, Trojanowska M. Endothelial cells recruit macrophages and contribute to a fibrotic milieu in bleomycin lung injury. Am J Respir Cell Mol Biol. 2013;49:1093–101. 23885794 10.1165/rcmb.2013-0152OC PMC3931119

[29] Kolb M, Margetts PJ, Anthony DC, Pitossi F, Gauldie J. Transient expression of IL-1beta induces acute lung injury and chronic repair leading to pulmonary fibrosis. J Clin Invest. 2001;107:1529–36. 11413160 10.1172/JCI12568 PMC200196

[30] Ciminieri C, Woest ME, Reynaert NL, Heijink IH, Wardenaar R, Spierings DCJ, Brandsma CA, Konigshoff M, Gosens R. IL-1beta induces a Proinflammatory fibroblast microenvironment that impairs lung progenitors’ function. Am J Respir Cell Mol Biol. 2023;68:444–55. 36608844 10.1165/rcmb.2022-0209OC PMC12042164

[31] Oikonomou N, Mouratis MA, Tzouvelekis A, Kaffe E, Valavanis C, Vilaras G, Karameris A, Prestwich GD, Bouros D, Aidinis V. Pulmonary autotaxin expression contributes to the pathogenesis of pulmonary fibrosis. Am J Respir Cell Mol Biol. 2012;47:566–74. 22744859 10.1165/rcmb.2012-0004OC

[32] Zhang Y, Lee TC, Guillemin B, Yu MC, Rom WN. Enhanced IL-1 beta and tumor necrosis factor-alpha release and messenger RNA expression in macrophages from idiopathic pulmonary fibrosis or after asbestos exposure. J Immunol. 1993;150:4188–96. 8473757

[33] Kline JN, Schwartz DA, Monick MM, Floerchinger CS, Hunninghake GW. Relative release of interleukin-1 beta and interleukin-1 receptor antagonist by alveolar macrophages. A study in asbestos-induced lung disease, sarcoidosis, and idiopathic pulmonary fibrosis. Chest. 1993;104:47–53. 8325116 10.1378/chest.104.1.47

[34] Lee CH, Sapkota A, Gaire BP, Choi JW. NLRP3 inflammasome activation is involved in LPA(1)-Mediated brain injury after transient focal cerebral ischemia. Int J Mol Sci. 2020;21(22). 10.3390/ijms21228595 PMC7697439 33202644

[35] Lendeckel U, Venz S, Wolke C. Macrophages: shapes and functions. ChemTexts. 2022;8:12. 35287314 10.1007/s40828-022-00163-4 PMC8907910

[36] Decato BE, Leeming DJ, Sand JMB, Fischer A, Du S, Palmer SM, Karsdal M, Luo Y, Minnich A. LPA(1) antagonist BMS-986020 changes collagen dynamics and exerts antifibrotic effects in vitro and in patients with idiopathic pulmonary fibrosis. Respir Res. 2022;23:61. 35303880 10.1186/s12931-022-01980-4 PMC8933988

[37] Xu MY, Porte J, Knox AJ, Weinreb PH, Maher TM, Violette SM, McAnulty RJ, Sheppard D, Jenkins G. Lysophosphatidic acid induces alphavbeta6 integrin-mediated TGF-beta activation via the LPA2 receptor and the small G protein G alpha(q). Am J Pathol. 2009;174:1264–79. 19147812 10.2353/ajpath.2009.080160 PMC2671359

[38] Alsafadi HN, Staab-Weijnitz CA, Lehmann M, Lindner M, Peschel B, Konigshoff M, Wagner DE. An ex vivo model to induce early fibrosis-like changes in human precision-cut lung slices. Am J Physiol Lung Cell Mol Physiol. 2017;312:L896–902. 28314802 10.1152/ajplung.00084.2017

[39] Palmer SM, Snyder L, Todd JL, Soule B, Christian R, Anstrom K, Luo Y, Gagnon R, Rosen G. Randomized, Double-Blind, Placebo-Controlled, phase 2 trial of BMS-986020, a lysophosphatidic acid receptor antagonist for the treatment of idiopathic pulmonary fibrosis. Chest. 2018;154:1061–9. 30201408 10.1016/j.chest.2018.08.1058

[40] Misharin AV, Morales-Nebreda L, Reyfman PA, Cuda CM, Walter JM, McQuattie-Pimentel AC, Chen CI, Anekalla KR, Joshi N, Williams KJN, et al. Monocyte-derived alveolar macrophages drive lung fibrosis and persist in the lung over the life span. J Exp Med. 2017;214:2387–404. 28694385 10.1084/jem.20162152 PMC5551573

[41] Grant RA, Poor TA, Sichizya L, Diaz E, Bailey JI, Soni S, Senkow KJ, Perez-Leonor XG, Abdala-Valencia H, Lu Z, et al. Prolonged exposure to lung-derived cytokines is associated with activation of microglia in patients with COVID-19. JCI Insight. 2024;9(8). 10.1172/jci.insight.178859 PMC11141878 38502186

[42] Perea L, Bottier M, Cant E, Richardson H, Dicker AJ, Shuttleworth M, Giam YH, Abo-Leyah H, Finch S, Huang JT, et al. Airway IL-1beta is related to disease severity and mucociliary function in bronchiectasis. Eur Respir J. 2024;64(2). 10.1183/13993003.01966-2023 38811046

