# Supplementary material for: The LPAR1 antagonist, PIPE-791 produces antifibrotic effects in models of lung fibrosis
Source: Respir Res. 2025 Aug 31;26:265. doi: 10.1186/s12931-025-03340-4 (PMC12400753; doi:10.1186/s12931-025-03340-4)
Supplement: Supplementary file 1 — Supplementary Material 1. [file 12931_2025_3340_MOESM1_ESM.docx]

**Reagent list**

| **Reagent** | **Catalog number** | **Vendor, Location** |
| --- | --- | --- |
| Antibiotic-Antimytotic | 15240-062 | Invitrogen, Carlsbad, CA |
| Aqua-poly/mount | 1860620 | Polysciences, Warrington, PA |
| aSMA antbody | 551351AP | Proteintech, Rosemont, IL |
| BI 1015550 |  | MedChemExpress, Monmouth Junction, NJ |
| Bleomycin | NDC-71288-106-10 | Meithewal, Chicago, IL |
| CD68 antibody | MCA341GA | Bio-rad, Herculues, CA |
| CellStripper | 25-056-CI | Corning, Corning, NY |
| Chemotaxis plate | CBA106 | Cell Biolabs, San Diego, CA |
| COL1A1 antibody | PA5-95137 | Thermo Fisher, Hercules CA |
| DC Protein Assay | 5000111 | Bio-rad, Herculues, CA |
| DMEM/F12 | 30-21006 | ATCC, Manassas, VA |
| ELISA MAX mouse IL-1b ELISA | 432601 | Biolegend, San Diego, CA |
| Fluo-4 NW | F36206 | Invitrogen, Carlsbad, CA |
| Goat anti mouse Alexa 488 antibody | A28175 | Invitrogen, Carlsbad, CA |
| Goat anti rabbit Alexa647 | A48265 | Invitrogen, Carlsbad, CA |
| Goat anti rat Alexa 488 | A-11034 | Invitrogen, Carlsbad, CA |
| Histamine EIA kit | EA31 | Oxford Biomedical Research, Rochester Hills, MI |
| Hoechst 33342 | H21492 | Invitrogen, Carlsbad, CA |
| Human Alveolar Macrophage Medium | ABM-H0034X | Accegen, Fairfield, NJ |
| Human alveolar macrophages | ABC-H0034X | Accegen, Fairfield, NJ |
| Human IL-1b ELISA | BMS2242 | Invitrogen, Carlsbad, CA |
| LPA (18:1) | 857130C | Avanti Polar Lipids, Alabaster AL |
| Lipopolysacchride | AX-100-012-M001 | Innaxon, San Diego, CA |
| OCT compound | 4583 | Tissue-Tek, Torrance, CA |
| Penicillin/Streptomycin | P4333 | Invitrogen, Carlsbad, CA |
| Perfecta SYBR Green FastMix | 95072 | Quantabio, Beverly, MA |
| PLN-74809 |  | Accela, San Diego, CA |
| Poly-L-lysine | A-005-C | Millipore Sigma, St Louis MO |
| Procollagen ELISA | ab210579 | Abcam, Waltham, MA |
| qScript XLT cDNA SuperMix | 95161 | Quantabio, Beverly, MA |
| Quickzyme Total Collagen kit |  | Quickzyme Bioscience, Leiden, Netherlands |
| RNEasy Mini | 74106 | Qiagen, Germantown, MD |
| TGFb ELISA | 100-21C | Invitrogen, Carlsbad, CA |
| TGFb ELISA | DB100C | R&D Systems, Minneapolis, MN |
| TIMP-1 ELISA | MTM100 | R&D Systems, Minneapolis, MN |
| Triton X 100 | X100 | Millipore Sigma, St Louis MO |
| Ultima Gold F | 50-905-0520 | Revvity, Waltham, MA |

| **Plasmid** | **Origene catalog number** |
| --- | --- |
| LPA1 | EDG20200000-02 |
| LPA2 | EDG0400000-02 |
| LPA3 | EDG0700000-02 |
| LPA4 | P2RY900000-03 |
| LPA5 | LPAR500000-03.1 |
| LPA6 | LPAR00000-02 |
|  |  |
| **Gene name** | **Primer sequence 5' -> 3'** |
| hCol1a1F | GATTCCCTGGACCTAAAGGTGC |
| hCol1a1R | AGCCTCTCCATCTTTGCCAGCA |
| hTNFaF | CTCTTCTGCCTGCTGCACTTTG |
| hTNFaR | ATGGGCTACAGGCTTGTCACTC |
| hPai1F | CTCATCAGCCACTGGAAAGGCA |
| hPai1R | GACTCGTGAAGTCAGCCTGAAAC |
| hCol3a1F | TGGTCTGCAAGGAATGCCTGGA |
| hCol3a1R | TCTTTCCCTGGGACACCATCAG |
| hTimp1F | GGAGAGTGTCTGCGGATACTTC |
| hTimp1R | GCAGGTAGTGATGTGCAAGAGTC |
| hEdg2 F | GGCTATGTTCGCCAGAGGACTA |
| hEdg2 R | GGAGTCCAGCAGATGATAAAGGC |
| h18S F | GTCTGTGATGCCCTTAGATG |
| h18S R | AGCTTATGACCCGCACTTAC |
