# Supplementary material for: The LPAR1 antagonist, PIPE-791 produces antifibrotic effects in models of lung fibrosis
Source: Respir Res. 2025 Aug 31;26:265. doi: 10.1186/s12931-025-03340-4 (PMC12400753; doi:10.1186/s12931-025-03340-4)
Supplement: Supplementary file 3 — Supplementary Material 3. [file 12931_2025_3340_MOESM3_ESM.pdf]

Supplemental Figures

Figure S1

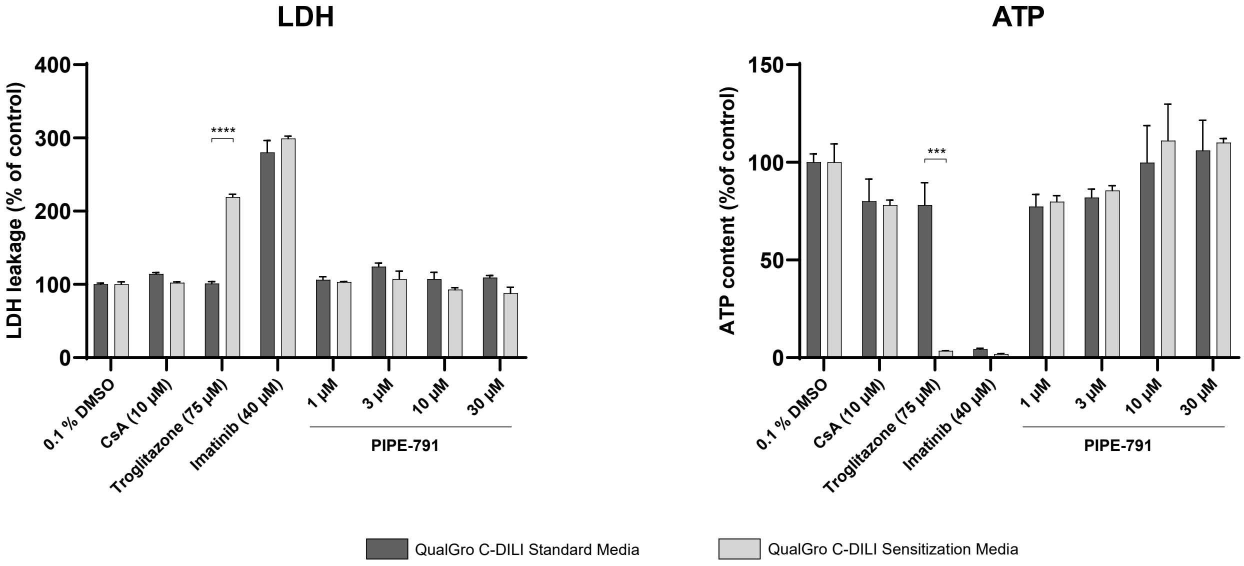

Transporter	Marker Substrate	PIPE-791 IC_{50} Value
BSEP	Taurochlorate (1 µM)	24.4 µM

Figure S1. PIPE-791 shows no potential for general and cholestatic hepatotoxicity (n=3, Student's t-test, *** P < 0.001, **** P < 0.0001, error bars are SD). Left, lactate dehydrogenase leakage, right, ATP content. Table shows minimal inhibition of BSEP.

Figure S2

Human primary adult lung fibroblast (COL1A1)

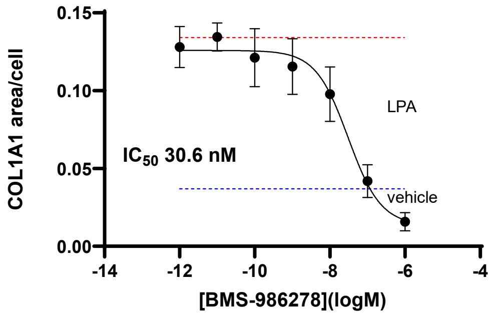

Figure S2. LPAR1 antagonist, BMS-986278 (admilparant) inhibits COL1A1 expression in normal human lung fibroblasts stimulated with LPA at an IC_{50} of 30.6 nM as assessed by immunocytochemistry (error bars are SEM).

Figure S3

A. B. C.

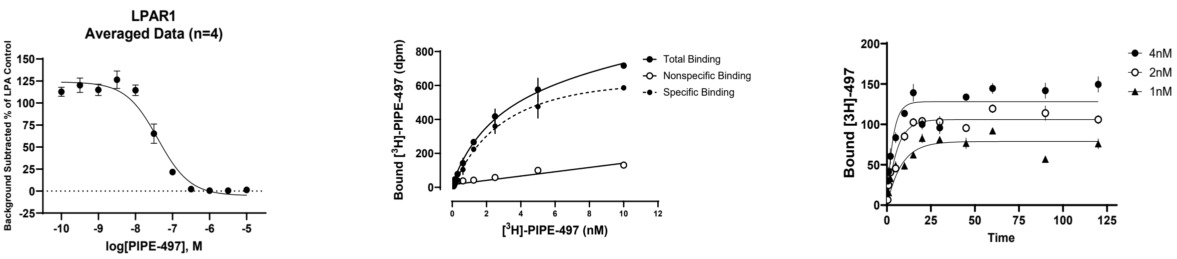

Figure S3. PIPE-497 is a potent LPAR1 receptor antagonist with properties suitable as an *in vivo* radioligand. A. LPAR1 calcium mobilization data showing that PIPE-497 (non-tritiated) is a potent LPAR1 receptor antagonist with an IC_{50} of 40 nM. B. Saturation binding curves of ^{3}H-PIPE-497 to human LPAR1 membranes (n=2, error bars are SEM). C. Binding kinetics of ^{3}H-PIPE-497 in recombinant membranes. Three concentrations of [^{3}H]-PIPE-497 (1, 2, and 4 nM) were incubated with 10 μM PIPE-497 then added to membranes at different time points resulting in a residence time of 16.2 minutes (n=4, error bars are SEM).

Figure S4

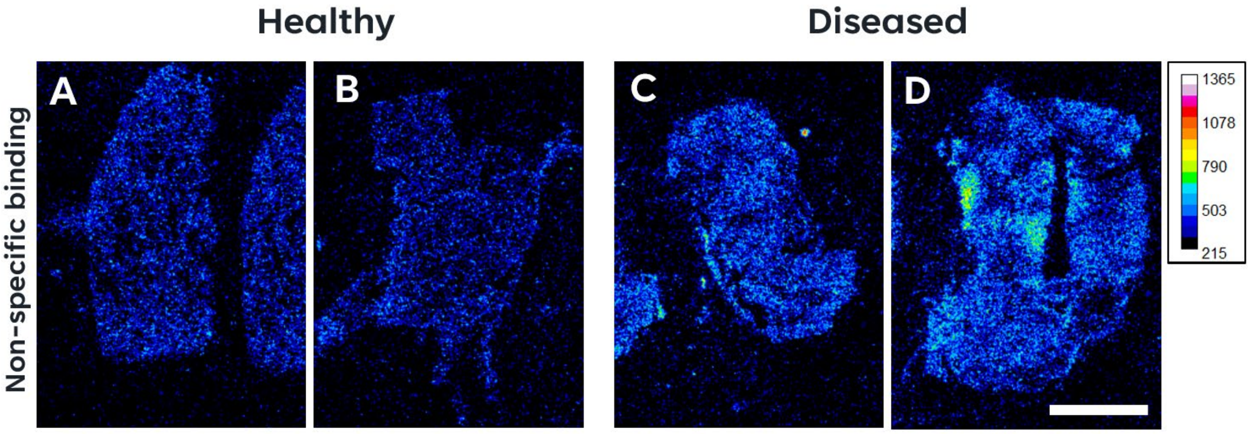

Figure S4. Representative images illustrating non-specific binding on human lung sections from healthy (A, B) and diseased donors (C, D). Signal intensity scale of the relative optical density is shown on the top right corner. Scale bar = 0.5 mm. Donor information is in Additional File 1.

Figure S5

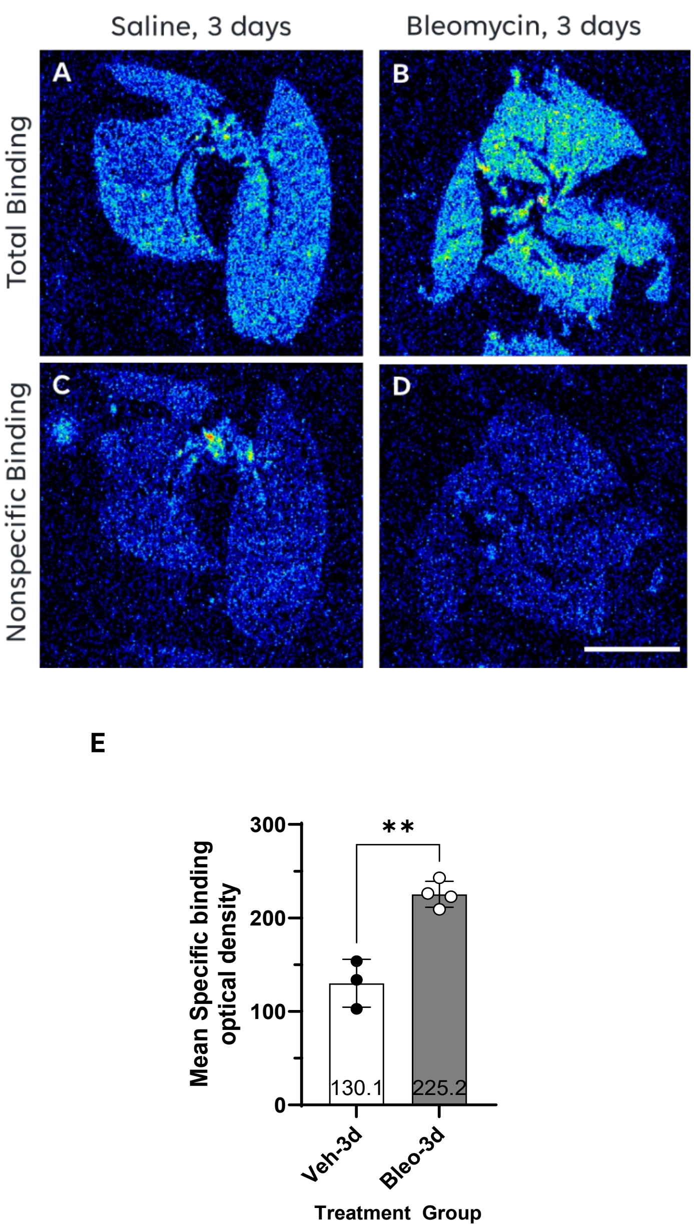

Figure S5. A-D. Representative images illustrating total (A, B) and non-specific (C, D) binding on mouse lung sections 3 days post oropharyngeal instillation of saline (A, C) or bleomycin (B, D). E. Mean lung specific binding was 1.7-fold increase in mice treated with bleomycin. (t-test, ** $P < 0.01$, error bars are SD) Scale bar = 0.5 mm.

Figure S6

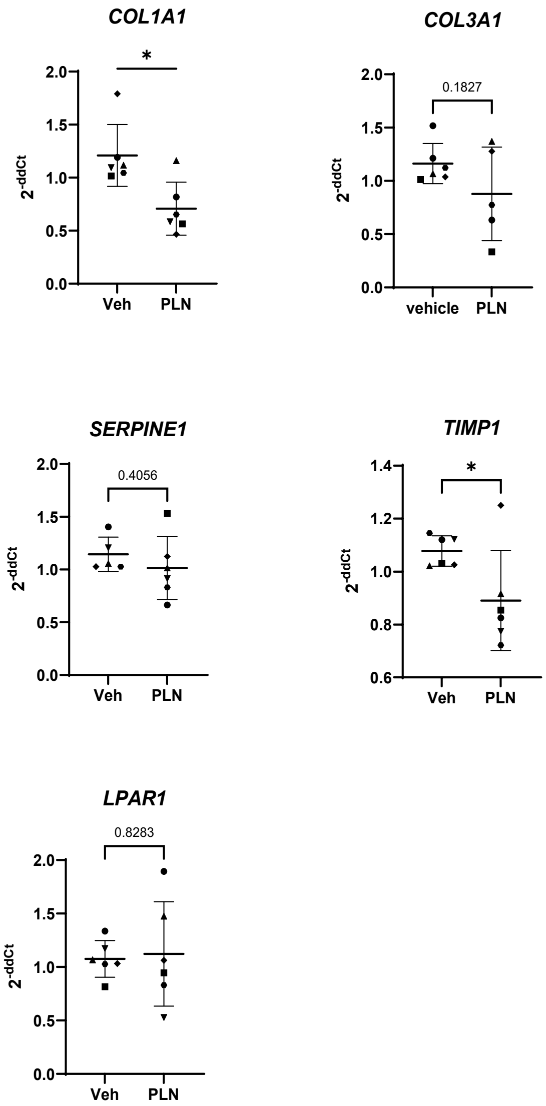

Figure S6. An $\alpha V\beta 1/6$ dual inhibitor, (PLN, PLN-74809, bexotegrast, 300 nM), reduces expression of fibrotic markers in PF PCLS including *COL1A1*, *COL3A1, SERPINE1, and TIMP1*. *LPAR1* expression is unchanged (n=6, t-test, * $P < 0.05$, P values listed above bars, error bars are SD). Symbols correspond to donors in Additional File 1 as follows: ● Donor G; ■ Donor H; ▲ Donor I; ◆ Donor J; ⬢ Donor K; ▼ Donor L.

Figure S7

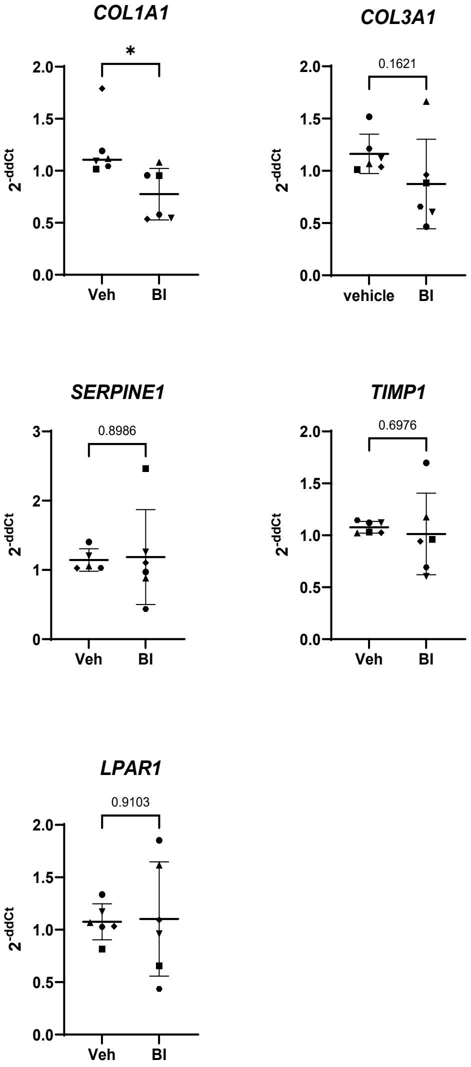

Figure S7. A PDE4B inhibitor (BI, BI 1015550, nerandomilast, 10 μM), reduces expression of fibrotic markers in PF PCLS including *COL1A1*, *COL3A1, TIMP1,* and *SERPINE1*. *LPAR1* expression is not significantly changed (n=6, t-test, * $P < 0.05$ or listed above bars, error bars are SD). Symbols correspond to donors in Additional File 1 as follows: ● Donor G; ■ Donor H; ▲ Donor I; ◆ Donor J; ⬣ Donor K; ▼ Donor L.

Figure S8

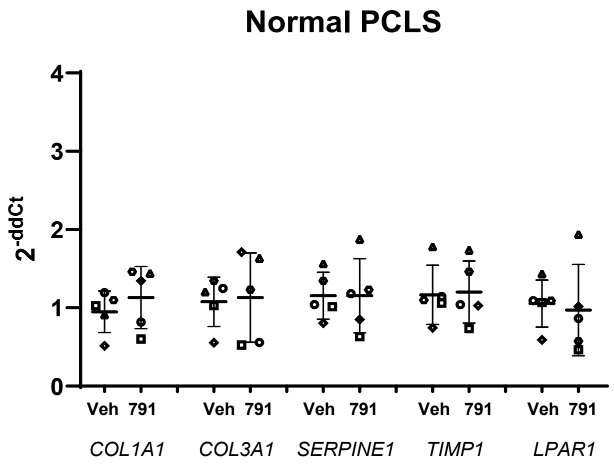

Figure S8. Treatment of normal lung PCLS with PIPE-791 does not significantly impact the expression of any fibrotic markers tested including *COL1A1*, *COL3A1, TIMP1, SERPINE, and LPAR1* (n=5, t-test, error bars are SD). Symbols correspond to donors as follows : ☐ Donor M; ○ Donor N; ◇Donor O; △ Donor P; ⬡ Donor Q.

Figure S9

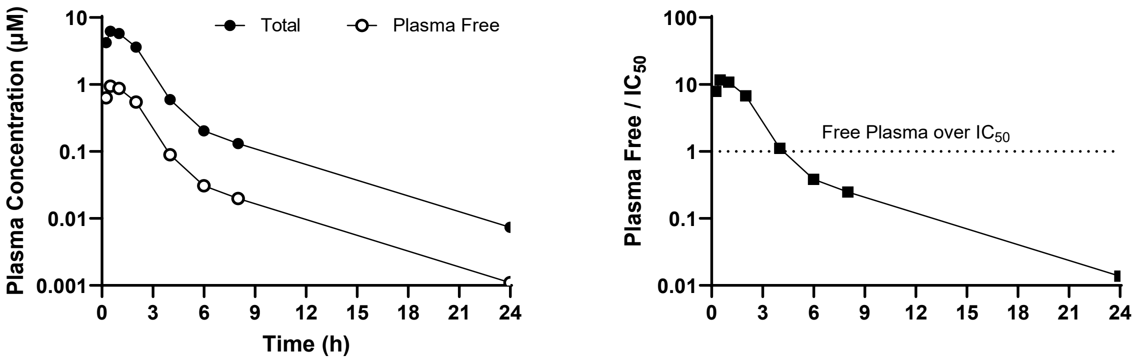

Figure S9. LPAR1 antagonist BMS-986278 covers IC_{50} until 4 h. Rats were orally dosed with BMS-986278 (10 mg/kg) and plasma concentrations taken at 0.25, 0.5, 1, 2, 4, 6, 8, and 24 hours post dose. Total (solid circles) and free plasma concentrations (open circles), n = 2. B. Graph depicting BMS-986278 plasma free (unbound) concentration as a function of time over the *in vitro* calcium mobilization IC_{50}. At 10 mg/kg, the free plasma exposure of BMS-986278 exceeds its measured IC_{50} up until 4 h post dose. IC_{50} in LPAR1 calcium mobilization was 80.4 ×/÷ 1.58 (n=6).

Supp Table 1

Parameter	Value
**K_{off} (min^{-1})**	0.06184
**Residence time (min)[a]**	16.2
**Residence time (h)[b]**	0.27
**$T_{1/2}$ (min)[c]**	11.2
**$T_{1/2}$ (h)[d]**	0.19
**[^{3}H]-PIPE-497 (nM)**	1, 2, 4
**B_{max} (dpm)**	161.9
**K_d (nM)**	1.09

[a] Residence Time (min) = 1/[K_{off} (min^{-1})]

[b] Residence Time (h) = K_{off} (min)/60

[c] $T_{1/2}$ (min) = Residence time (min) x 0.693 = 0.693/K_{off} (min^{-1})

[d] $T_{1/2}$ (h) = [$T_{1/2}$ (min)]/60

Supp Table 1. Summary table of properties of [^{3}H]-PIPE-497.
